# A *Plasmodium berghei* sporozoite-based vaccination platform against human malaria

**DOI:** 10.1038/s41541-018-0068-2

**Published:** 2018-08-24

**Authors:** António M. Mendes, Marta Machado, Nataniel Gonçalves-Rosa, Isaie J. Reuling, Lander Foquet, Cláudia Marques, Ahmed M. Salman, Annie S. P. Yang, Kara A. Moser, Ankit Dwivedi, Cornelus C. Hermsen, Belén Jiménez-Díaz, Sara Viera, Jorge M. Santos, Inês Albuquerque, Sangeeta N. Bhatia, John Bial, Iñigo Angulo-Barturen, Joana C. Silva, Geert Leroux-Roels, Chris J. Janse, Shahid M. Khan, Maria M. Mota, Robert W. Sauerwein, Miguel Prudêncio

**Affiliations:** 1 Instituto de Medicina Molecular, Faculdade de Medicina, Universidade de Lisboa, Avenida Professor Egas Moniz, 1649−028 Lisboa, Portugal; 2 Department of Medical Microbiology, Radboud University Medical Center, Geert Grooteplein 28, Microbiology 268, 6500 HB Nijmegen, The Netherlands; 3 Center for Vaccinology, Ghent University and Ghent University Hospital, De Pintelaan 185, 9000 Ghent, Belgium; 4 Departments of Clinical Chemistry, Microbiology and Immunology, Ghent University, Ghent University Hospital, Ghent, Belgium; 5 Leiden Malaria Research Group, Parasitology, Center of Infectious Diseases, Leiden University Medical Center, Leiden, The Netherlands; 6 The Jenner Institute, Nuffield Department of Medicine, University of Oxford, ORCRB, Roosevelt Drive, Oxford, OX3 7DQ UK; 7 Institute for Genome Sciences, University of Maryland School of Medicine, Baltimore, MD 21201 USA; 8 Diseases of the Developing World, GlaxoSmithKline, Severo Ochoa, 2, 28760 Tres Cantos, Madrid Spain; 9 Health Sciences and Technology/Institute for Medical Engineering and Science, Massachusetts Institute of Technology, Cambridge, MA 02139 USA; 10 Yecuris Corporation, PO Box 4645, Tualatin, OR 97062 USA; 11 Department of Microbiology and Immunology, University of Maryland School of Medicine, Baltimore, MD 21201 USA; 12 Present Address: Department of Immunology and Infectious Diseases, Harvard T.H. Chan School of Public Health, 665 Huntington Avenue, 02115 Boston, MA USA

## Abstract

There is a pressing need for safe and highly effective *Plasmodium falciparum* (*Pf*) malaria vaccines. The circumsporozoite protein (CS), expressed on sporozoites and during early hepatic stages, is a leading target vaccine candidate, but clinical efficacy has been modest so far. Conversely, whole-sporozoite (WSp) vaccines have consistently shown high levels of sterilizing immunity and constitute a promising approach to effective immunization against malaria. Here, we describe a novel WSp malaria vaccine that employs transgenic sporozoites of rodent *P. berghei* (*Pb*) parasites as cross-species immunizing agents and as platforms for expression and delivery of *Pf*CS (*Pb*Vac). We show that both wild-type *Pb* and *Pb*Vac sporozoites unabatedly infect and develop in human hepatocytes while unable to establish an infection in human red blood cells. In a rabbit model, similarly susceptible to *Pb* hepatic but not blood infection, we show that *Pb*Vac elicits cross-species cellular immune responses, as well as *Pf*CS-specific antibodies that efficiently inhibit *Pf* sporozoite liver invasion in human hepatocytes and in mice with humanized livers. Thus, *Pb*Vac is safe and induces functional immune responses in preclinical studies, warranting clinical testing and development.

## Malaria: Programming non-pathogenic parasites as vaccine candidates

A genetically engineered parasite, related to malaria-causing *Plasmodium falciparum*, excels as a vaccine in preclinical tests. A team led by Miguel Prudêncio, of the University of Lisbon, Portugal, developed a genetically altered vaccine candidate based on *Plasmodium berghei*, which is pathogenic to rodents but, in humans, fails to progress from a harmless, transient liver infection to causing full, blood-borne malaria. The candidate expresses a human form of ‘circumsporozoite protein,’ a known antigen, and is designed to provoke a more comprehensive immune response as it presents a whole pathogen to the host. In preclinical tests, the candidate generated antibodies able to neutralize infection in human hepatocytes and also provoked a cellular immune response in rabbits. The team’s candidate proved safe and efficacious, warranting further trials and clinical testing.

## Introduction

The long-standing goal of an effective vaccine against malaria constitutes a crucial component of efforts to prevent a disease that continues to kill nearly half a million people per year.^1^ During a natural malaria infection, *Plasmodium* sporozoites are injected into the skin and skin vasculature by an infected mosquito and travel to the liver of their vertebrate host. An asymptomatic parasite maturation and replication phase inside hepatocytes ensues, leading to the generation of *Plasmodium* exoerythrocytic forms (EEFs) and preceding the release of erythrocyte-infectious merozoites, which can establish a blood infection and lead to disease symptoms [reviewed in^2^].

So far, vaccines against the early pre-erythrocytic stages of *Plasmodium* parasites have shown most success among current vaccine candidates,^3^ including the most advanced subunit vaccine against the human malaria parasite *P. falciparum* (*Pf*), RTS,S, that targets the circumsporozoite (CS) protein,^4^ the predominant antigen on the surface of sporozoites and a major vaccine candidate. While the ability of CS-based vaccination to partially limit clinical malaria infection in the field is a major achievement, the modest and rapidly waning efficacy of RTS,S stresses the urgency to develop vaccines with higher and more durable protection.^5^ An alternative to subunit vaccines is the use of whole-sporozoite (WSp) approaches, based on the generation of immunity against *Plasmodium* pre-erythrocytic stages following immunisation with infective sporozoites under conditions that prevent the appearance of clinical symptoms, including radiation-attenuated sporozoites (RAS),^6–8^ genetically attenuated parasites (GAP),^9–13^ and immunisation with non-attenuated sporozoites in combination with chemoprophylaxis (CPS).^14–16^ Although CS has been proposed to play an important protective role in WSp vaccines, complete protection following *P. yoelii* RAS immunization has been shown to occur in transgenic mice that are T-cell tolerant to CS and cannot produce antibodies.^17^ Therefore, protection induced by WSp is likely mediated by a plethora of hitherto unidentified liver stage antigens presented to the immune system during liver stage parasite development (reviewed in^18^). Accordingly, later liver stage-arresting parasites, such as some GAP parasites, and those completing liver stage development, such as the CPS approach, seem to trigger antimalarial immunity superior to that elicited by early-arresting variants.^11,19^ Nonetheless, the most advanced WSp approach to human vaccination relies on the intravenous administration of the PfSPZ Vaccine, composed of aseptic, purified, cryopreserved *Pf*RAS.^20–23^ While all current WSp human vaccination strategies rely on the use of *Pf* sporozoites, alternative WSp vaccines can also be envisaged. In this context, a rodent *Plasmodium*-based immunization platform constitutes an inherently safe and hitherto unexplored approach to WSp vaccination that is worth investigating.

The paradigm of immunizing with non-pathogenic microorganisms to protect against the disease caused by their human-infective counterparts was pioneered by Edward Jenner using a bovine poxvirus to prevent smallpox. This concept of vaccination has since been employed for various other human diseases, through the development of the bovine bacillus Calmette–Guérin (BCG) vaccine against human TB or the selection of rhesus and bovine rotavirus strains to create human rotavirus vaccines (reviewed in^24^). Vaccine development has also benefitted from advances in genetic manipulation, which have allowed for isolation, modification, and optimization of vaccine antigen delivery and facilitated smart vaccine design. The first genetically modified (GM) human vaccine, against hepatitis B, was approved in 1986,^25^ followed by vaccines targeting influenza^26^ and Japanese encephalitis.^27^ However, the combination of cross-species immunity and genetic modification has never been applied to the field of malaria vaccination.

We propose to use GM rodent *Plasmodium* sporozoites expressing human *Plasmodium* antigens as a safe “naturally attenuated” WSp vaccination platform that can elicit cross-species immune responses against *Pf* as well as deliver specific immunogens, such as *Pf*CS, and that may protect against a subsequent infection by human malaria parasites. Rodent *P. berghei* (*Pb*) parasites efficiently infect human hepatocytes in vivo, a requirement for optimal antigen presentation, while remaining unable to cause a blood-stage infection, in agreement with the widely accepted notion that they are non-pathogenic to humans. We establish the proof-of-principle of this vaccination approach by demonstrating that immunisation of rabbits with transgenic *Pb* sporozoites expressing *Pf*CS (*Pb*Vac) induces robust immunity against *Pf*, including cross-species cellular immune responses, as well as *Pf*CS-dependent humoral responses that functionally block *Pf* infection of liver-humanized mice. These results identify a new *Pb*-based WSp immunizing agent and antigen delivery platform for malaria vaccination and pave the way for the design of rodent parasites that can induce optimal protective immune responses against human malaria.

## Results

### *P. berghei* can infect human hepatocytes but is unable to develop in human erythrocytes

**Fig. 1:**
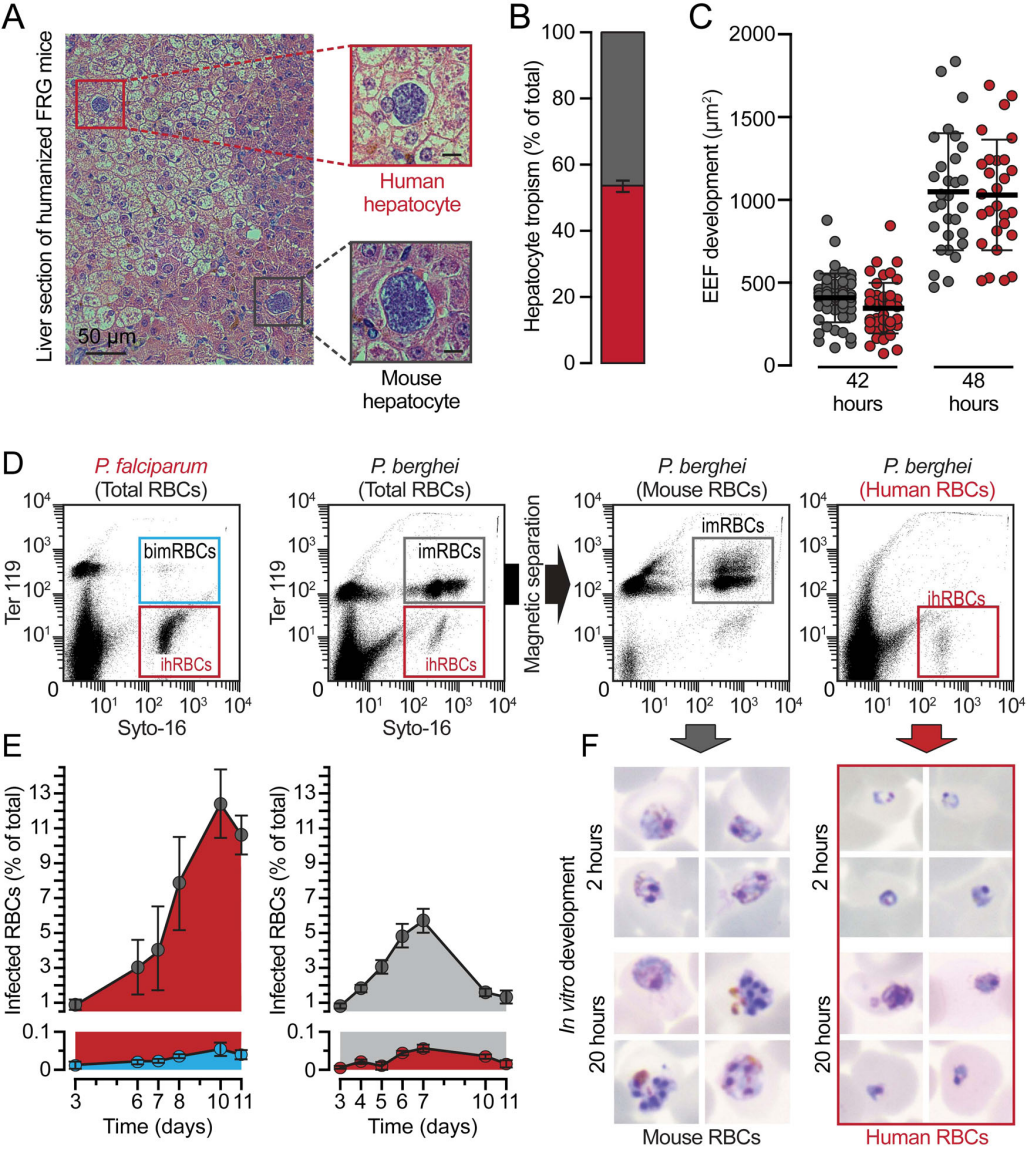
Rodent *P. berghei* parasites successfully develop within human hepatocytes but not within human RBCs. **a** Representative images of developing rodent *Pb*WT parasites in mouse (black square) and human (red square) hepatic cells of liver-humanized FRG mice 48 h post infection (hpi) by iv injection of freshly isolated sporozoites. **b** Relative proportion of *Pb*-infected mouse (grey) and human (red) hepatocytes in humanized FRG mice, normalized to the total humanization of the chimeric liver. **c** *Pb*WT development in mouse (grey) and human (red) hepatocytes 42 and 48 hpi of liver-humanized FRG mice. **d** Representative flow cytometry plots of peripheral blood from blood-humanized NSG mice infected by iv injection of *Pf*-infected (left) or *Pb*-infected RBCs (middle-left) before and after magnetic separation (middle-right and right); Syto-16 for nucleic acids; TER-119 for murine erythroid lineage; imRBCs/ihRBCs: infected mouse or human RBCs; bimRBCs: background signal for infected murine erythroid lineage. **e** Relative proportion of mouse and human RBCs infected with *Pf* (left) or *Pb* (right) parasites; bars indicate standard error. **f** Representative pictures of *Pb* parasite forms observed within magnetically separated imRBCs and ihRBCs from the total blood of infected blood-humanized NSG mice after 2 and 20 h of in vitro culture

It is well known that the sporozoite stage of *Pb* is able to infect hepatic cells from different hosts, including several human-derived and mouse-derived hepatoma cell lines and human primary hepatocytes (PH) cultured ex vivo [reviewed in^28^]. We confirmed and extended these findings by monitoring in parallel the in vitro infection of one mouse and two human hepatoma cell lines (Hepa 1-6, HepG2, and Huh7, respectively), and one human immortalized hepatocyte line (HC-04), as well as the ex vivo infection of human PH/fibroblast co-cultures by *Pb*. Infection assessed by immunofluorescence microscopy showed that sporozoites invade and develop to similar extents in all in vitro systems studied (Fig. S1A-C) and that these parasites are able to invade and develop inside human PH ex vivo (Fig. S1D-F). We further ascertained *Pb* infectivity of human hepatocytes in vivo, in liver-humanized FRG mice. Our results show that *Pb* can effectively infect human hepatocytes engrafted in liver-humanized FRG mice (Fig. 1a and Fig. S1G, H), displaying similar tropism to mouse and human hepatocytes (Fig. 1b), and similar development inside either type of cell (Fig. 1c and Fig. S1H).

We subsequently assessed *Pb* infectivity of human red blood cells (RBC) employing blood-humanized mice, engrafted with defined proportions of human RBC,^29^ infected by transfusion of infected RBC. Coupled use of nuclear SYTO-16 and mouse erythroid line-specific TER-119 dyes allowed the distinction between infected and non-infected cells and between human and rodent RBC, respectively, and thereby enabling monitoring of infection by flow cytometry (Fig. 1d). Our results show that while the SYTO-16^+^/TER-119^+^ population, indicative of infection of the mouse RBC population, increased steadily, the SYTO-16^+^/TER-119^−^ population, corresponding to infected human RBC, remained below 0.1%, similar to the background signal observed for mouse RBCs in *Pf*-infected blood-humanized mice (Fig. 1d and e). Fluorescence microscopy analysis of these samples revealed very few SYTO-16^+^/TER-119^−^ cells indicating the rare occurrence of invasion of human RBCs by *Pb* (data not shown). We could not find any human RBC bearing a parasite with more than a single nucleus, suggesting that the parasite degenerates into unviable cryptic forms. To ascertain this, infected human and mouse RBC were isolated by TER-119-based magnetic activated cell sorting and the isolated cells were cultured in vitro for up to 20 h. Our in vitro results show that while parasites in infected mouse RBC were able to develop as expected, parasites in the infected human RBC population remained single-nucleated and unable to multiply (Fig. 1f). Similar results were obtained when infection of blood-humanized mice was initiated by sporozoite injection (data not shown). Overall, these results clearly show that *Pb* is capable of infecting human hepatocytes whereas it is unable to develop inside human RBC.

### The *Pb*Vac vaccination platform

In order to assess the potential of *Pb* to elicit cross-species immune responses against *Pf*, a comprehensive, in silico prediction of CD8^+^ T cell epitopes in the proteomes of *Pf* and *Pb* was carried out. Our results show that 24171 in silico-predicted epitopes are shared between species. These are encoded in 61% (3371/5548) of the *Pf* proteins and 66% (3332/5059) *Pb* proteins, of which 3223 are orthologous pairs in the two species. This includes several antigens expressed during pre-erythrocytic stages (e.g., SLARP, SIAP1, LISP1, and MB2), and substantiates the potential for cross-protection between the two species (Fig. S2, Tables S1, S2). Notably absent from the set of *Pf* proteins containing shared epitopes with *Pb* is CS.

Given the established value of *Pf*CS as a leading vaccine candidate antigen, we generated a transgenic *Pb* line, *Pb*Vac, that expresses *Pf*CS, using ‘gene insertion/marker out’ (GIMO) methods of transfection.^30^ The gene encoding *Pf*CS was inserted into the neutral *230p* locus of the *Pb* genome under the control of the 5′- and 3′ regulatory sequences of *Pb*’s upregulated in infective sporozoites 4 (*uis4*) gene (Fig. S3A), which is expressed exclusively in sporozoites and developing liver stages.^31^ The GIMO transfection method employed ensures the stable insertion of the gene encoding the heterologous *Pf*CS and flanking regions in the *Pb* genome, resulting in a drug-selectable marker-free transgenic parasite.^30^ Genotyping of *Pb*Vac showed correct integration of the *Pf*CS expression cassette (Fig. S3B and C).

**Fig. 2:**
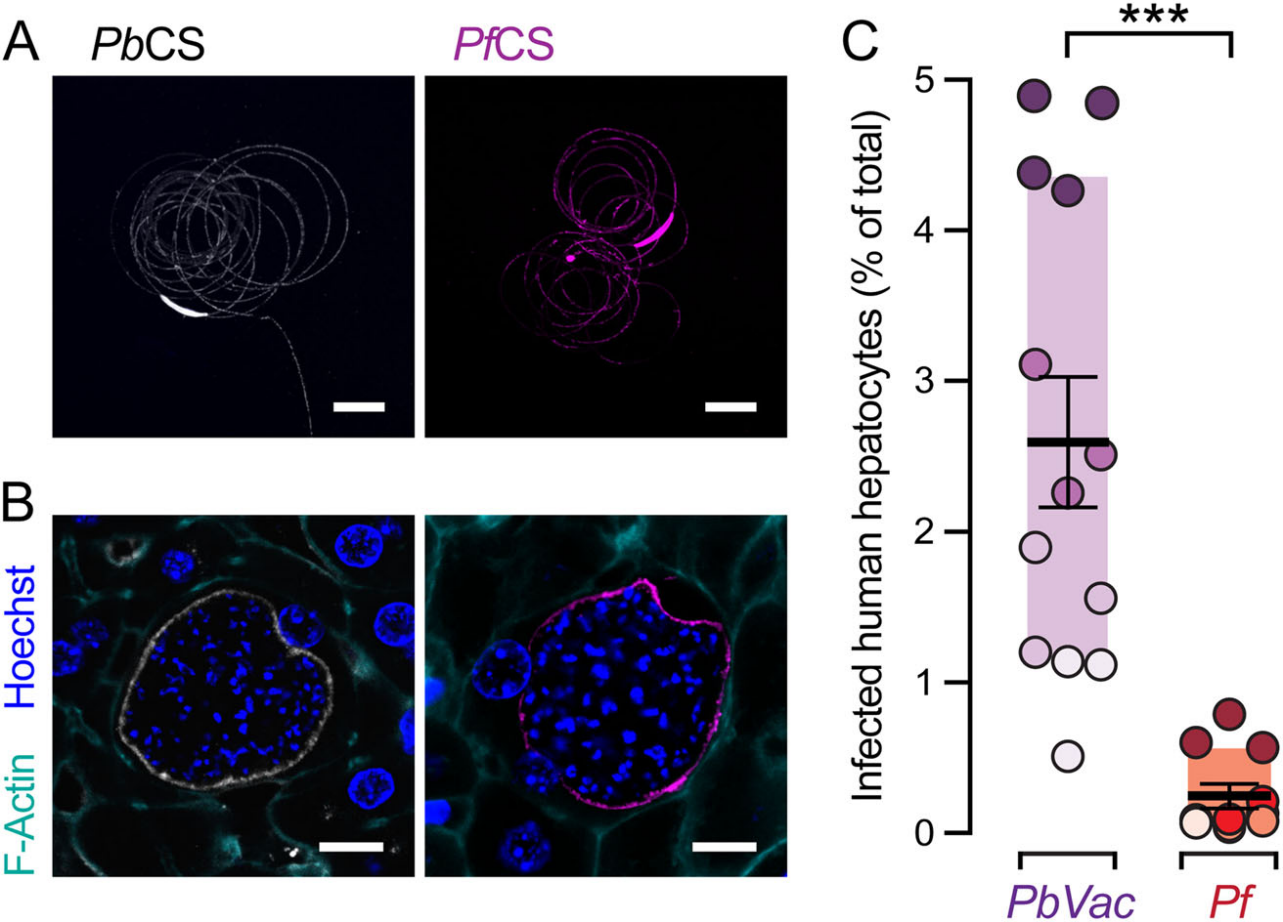
*Pf*CS expression and human hepatic infectivity of *Pb*Vac pre-erythrocytic stages. **a**, **b** Representative Immunofluorescence microscopy images of *Pb*CS (grey) and *Pf*CS (purple) expressed by *Pb*Vac sporozoites **a** and exoerythrocytic forms in the livers of mice infected by iv injection of freshly isolated sporozoites **b**. **c** Comparative infection rates of *Pb*Vac and *Pf* parasites in ex vivo cultures of human hepatocytes assessed by immunofluorescence microscopy. The shading of dots indicates distinct biological replicates obtained employing human hepatocytes from different donors. The boxes correspond to the 25th and 75th percentiles; the lines and bars indicate mean of infection and standard error of the mean, respectively; ***p < 0.001, as determined by Mann–Whitney *U* test. Scale bars: 10 µm

We next sought to assess the impact of genetic manipulation on the overall fitness of *Pb*Vac. To this end, we started by evaluating *Pb*Vac’s sporogonic development and showed that it was indistinguishable from that of the parental wild-type *Pb* (*Pb*WT), contrary to what was observed in previous attempts at expressing *Pf*CS as a replacement of the endogenous *Pb*CS gene.^32^ The two parasite lines formed similar numbers of oocysts in the mosquito host’s midgut (Fig. S4A), as well as of sporozoites in oocysts (Fig. S4B), in the hemolymph (Fig. S4C), and in salivary glands (Fig. S4D). We then analyzed the expression of the endogenous *Pb*CS and heterologous *Pf*CS proteins, in *Pb*Vac and *Pb*WT parasites. Immunofluorescence microscopy analysis clearly shows that only *Pb*CS is expressed and shed by *Pb*WT sporozoites (data not shown), while the *Pb*Vac sporozoites express and shed both the *Pb*CS and the *Pf*CS proteins during gliding (Fig. 2a). Our results further show that both proteins are expressed by developing *Pb*Vac parasites during hepatic development and are present at the parasite membrane both in in vivo (Fig. 2b) and ex vivo (Fig. S4E and F). We then compared the hepatic infectivity of *Pb*Vac and *Pb*WT sporozoites in mice. Immunofluorescence microscopy analysis of ex vivo-infected mouse PH revealed that both parasites yield equivalent numbers of EEFs (Fig. S4G), which have comparable development (Fig. S4H). Subsequent qRT-PCR and immunofluorescence microscopy analyses of infected mouse livers further confirmed that *Pb*Vac and *Pb*WT sporozoites lead to similar total hepatic parasite loads (Fig. S4I), with identical numbers of EEFs formed (Fig. S4J) and similar in vivo development (Fig. S4K). Additionally, we showed that, like *Pb*WT, the *Pb*Vac parasites readily infect human hepatocytes in liver-humanized FRG mice (Fig. S5A and B*)* but, unlike *Pf* parasites, are unable to multiply in human RBC, degenerating into unviable cryptic forms (Fig. S5C and D). Finally, we compared the infectivity of *Pb*Vac and *Pf* sporozoites to freshly isolated human PH ex vivo. Our data showed that the number of resulting EEFs in human PH, as determined by microscopy 48 h after sporozoite addition, can be up to 50-fold higher for *Pb*Vac than for *Pf* (Fig. 2c). Collectively, we show that *Pb*Vac parasites display similar fitness to *Pb*WT and that the engineered *Pf*CS protein is correctly expressed, localizing to the surface of *Pb*Vac sporozoites. Our results further show that *Pb*Vac parasites are unable to develop inside human erythrocytes and, most importantly, are able to infect human hepatocytes with even greater efficiency than *Pf*.

### An innovative animal model for evaluation of *Pb*Vac immunogenicity

**Fig. 3:**
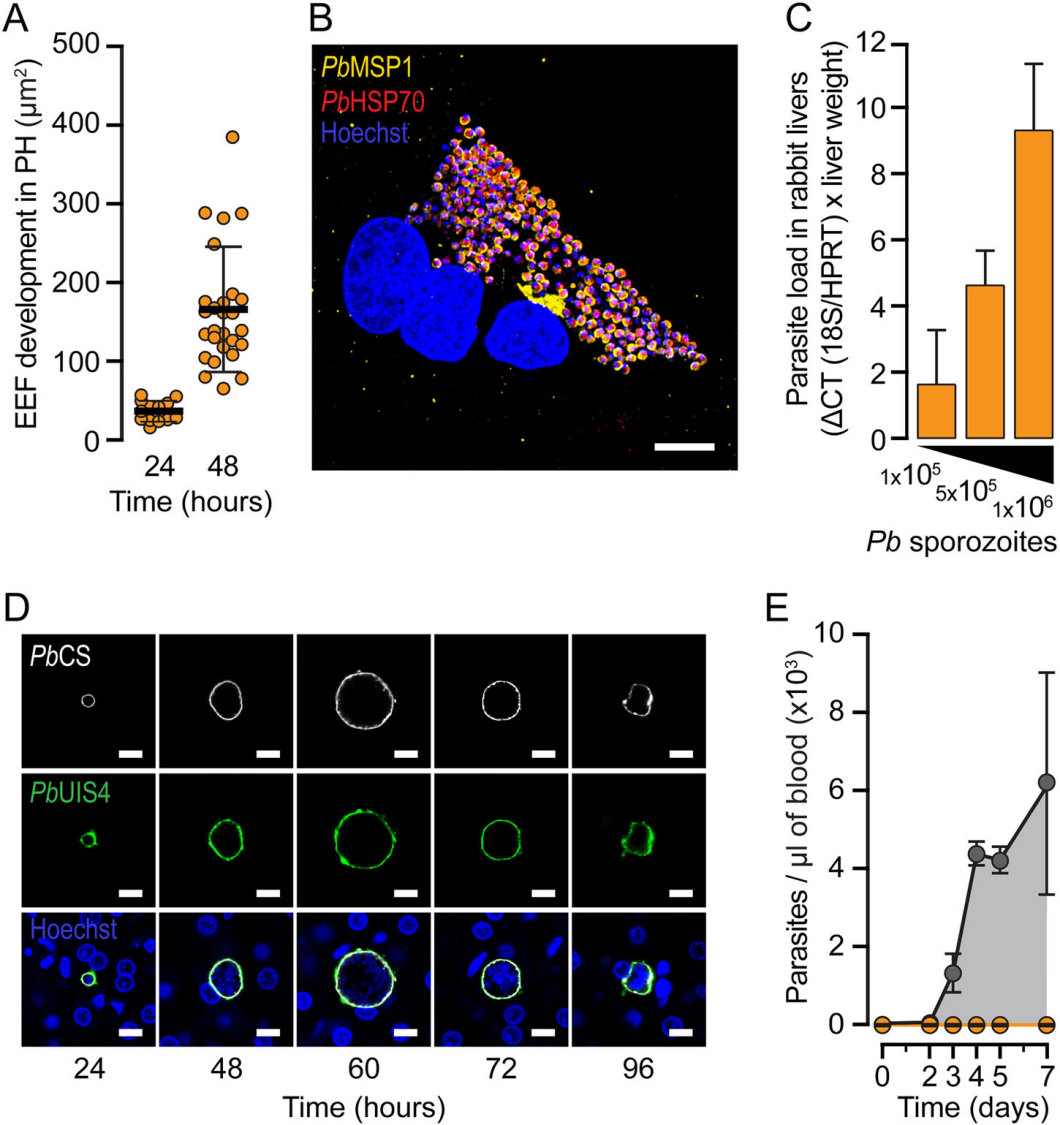
Infection of NZW rabbit hepatocytes with *P. berghei* sporozoites. **a** Rodent *Pb* parasite development 24 and 48 hpi in ex vivo cultures of rabbit primary hepatocytes. **b** Representative immunofluorescence microscopy image of a *Pb* merosomes developing ex vivo within rabbit primary hepatocytes 60 hpi and presenting typical markers of late exoerythrocytic development. **c** qRT-PCR quantification of hepatic infection of rabbits following iv injection of increasing amounts of freshly isolated *Pb* sporozoites (1 × 10^5^, 5 × 10^5^, and 1 × 10^6^). **d** Representative immunofluorescence microscopy images of rodent *Pb* parasites developing in the livers of NZW rabbits at different hpi. **e** Parasitemia in the peripheral blood of rabbits (orange) or mice (grey) following iv injection of freshly isolated *Pb* sporozoites. Scale bars: 10 µm

Having constructed and characterized the *Pb*Vac vaccine candidate, we then sought to assess its ability to elicit immune responses against human-infective *Pf* parasites. Rodents do not constitute an appropriate animal model in that respect, as they are susceptible to blood-stage infection by *Pb*Vac. To overcome this limitation, we evaluated New Zealand White (NZW) rabbits as an alternative model that mimics *Pb*’s pattern of infectivity in humans. To this end, we started by infecting NZW rabbit PH with *Pb* sporozoites and assessed infection at different time points after sporozoite addition by immunofluorescence microscopy. The results show that *Pb* effectively invades and develops inside rabbit PH (Fig. 3a and Fig. S6A) and is capable of completing its hepatic developmental process and forming infectious merozoites ex vivo (Fig. 3b and Fig. S6B). To ascertain *Pb*’s ability to infect rabbit hepatocytes in vivo, NZW rabbits were exposed to infected mosquito bites or increasing numbers of *Pb* sporozoites injected intravenously. qRT-PCR and detailed immunofluorescence microscopy analyses of rabbit livers showed that *Pb* readily infects rabbit hepatocytes in an in vivo context (Fig. 3c) developing for a longer period than the 58–62 h of development observed in highly susceptible hosts such as BALB/c or C57BL/6 mice. Our results further indicate that larger EEFs disappear from the rabbit livers earlier than less developed parasites, which can be observed up to 96 h after infection (Fig. 3d and Fig. S6C and D), therefore appearing to persist longer than irradiated parasites do in the liver of susceptible hosts (data not shown). Finally, to investigate rabbit RBC infectivity by *Pb*, NZW rabbits were infected with luciferase-expressing *Pb* sporozoites and blood samples were collected daily and analyzed by Giemsa staining and luminescence. As controls, mice were infected with similar numbers of *Pb* sporozoites and blood samples were collected at the same time points. Our results show that whereas parasitemia increased steadily in the blood of infected mice, no parasites were detected in the NZW rabbit blood up to 7 days after parasite administration (Fig. 3e). To fully establish the inability of *Pb* to infect NZW rabbit RBC, rabbits and mice were also infected by blood transfusion with 1 × 10^8^ *Pb*-infected mouse erythrocytes. Again, while parasitemia developed as expected in the blood of infected mice, no parasites were detected in rabbit blood (Fig. S6E) even following prolonged treatment with phenylhydrazine to increase reticulocytemia (Fig. S6F).

### Immune responses elicited by immunization with *Pb*Vac parasites

**Fig. 4:**
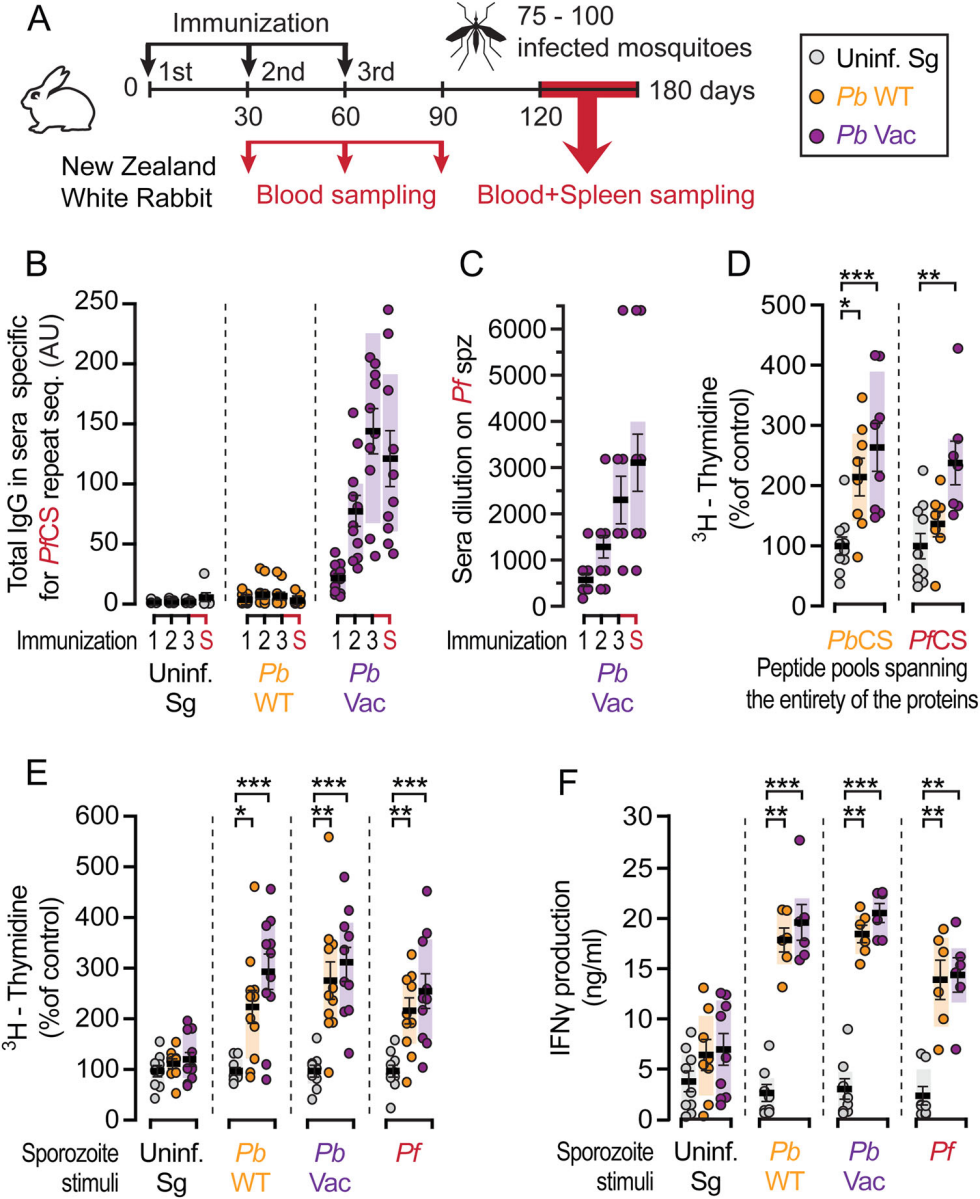
Immune responses in NZW rabbits after *Pb*Vac sporozoite immunization. **a** Diagram of the immunization protocol. Immunizations were performed by exposure to the bites of 75-100 mosquitoes. **b** Total IgG titers against *Pf*CS repeat sequence in serum after 1, 2, and 3 mock immunizations (grey), or immunization with *Pb*WT (orange) and *Pb*Vac (purple), or at the time of animal sacrifice (S) 60–90 days after last immunization. **c** Serum binding capacity to *Pf* sporozoites after *Pb*Vac immunization. **d** Spleen cell proliferation upon stimulation with peptide pools spanning the entire *Pb*CS or *Pf*CS proteins in immunized rabbits, as indicated by assessment of ^3^H-thymidine incorporation. **e** Spleen cell proliferation upon stimulation with *Pb*WT, *Pb*Vac or *Pf* sporozoites, as indicated by assessment of ^3^H-thymidine incorporation. Stimulation with an extract of uninfected mosquito salivary gland material was used as control. **f** IFNy production in rabbit spleen cell supernatant after stimulation with *Pb*WT, *Pb*Vac or *Pf* sporozoites. Measurements were taken from distinct samples. The boxes correspond to the 25th and 75th percentiles; the line and bars indicate mean of infection and standard error of the mean, respectively; *p < 0.05; **p < 0.01; ***p < 0.001, as determined by Kruskal–Wallis test, corrected with Dunn’s multiple comparisons test

Having shown that they constitute a model of *Pb* hepatic infection that can be employed in immunization studies with *Pb*-based sporozoite vaccines, NZW rabbits were immunized by repeated mosquito bite delivery of *Pb*WT or *Pb*Vac sporozoites for immune response assessment. In parallel, non-infected mosquitoes were allowed to feed on mock-immunized controls. Blood samples were collected at the time of the 2nd and 3rd immunizations, as well as one month after the 3rd and last immunization. Two to four months after the last of three immunizations, rabbit blood and splenocytes samples were collected for processing and subsequent analysis (Fig. 4a). We started by quantifying total IgGs in the serum of immunized animals by ELISA, employing peptides spanning the *Pf*CS repeat region. Anti-*Pf*CS antibody titers in the serum of *Pb*Vac-immunized animals steadily increased after each of the three immunizations, confirming “vaccine take” in these animals and successful delivery of the heterologous *Pf*CS antigen by *Pb*Vac (Fig. 4b). Anti-*Pb*CS antibody titers measured as controls in these experiments were also found to increase after each immunization with either *Pb*WT or *Pb*Vac, whereas neither anti-*Pf* CS nor anti-*Pb*CS antibodies were detected in mock-immunized animals (Fig. S7A). Having shown that *Pf*CS transgene presentation upon immunization with *Pb*Vac leads to a strong humoral response, we then investigated the responses elicited against whole *Pf* sporozoites. Indirect fluorescence antibody test (IFAT) analyses of immune sera collected at various time points revealed the presence of increasing amounts of antibodies that recognize and bind to immobilized *Pf* sporozoites in the serum of *Pb*Vac-immunized (Fig. 4c), but not in that of mock-immunized or *Pb*WT-immunized (Fig. S7B), rabbits. We then asked whether a CS-specific cellular immune response was also induced by immunization. To this end, rabbit splenic lymphocyte proliferation was assessed by a ^3^H-thymidine incorporation assay following stimulation with peptide pools spanning the entire amino acid sequence of either the *Pb*CS or the *Pf*CS proteins. A marked proliferation of lymphocytes of *Pb*WT-immunized rabbits was only observed in response to a *Pb*CS stimulus, whereas those of *Pb*Vac-immunized animals significantly proliferated upon stimulation with either *Pb*CS or *Pf*CS (Fig. 4d and Fig. S8). Of note, proliferation was not observed when the cells were stimulated with peptides spanning only the conserved repeat regions of the *Pb*CS or *Pf*CS proteins (Fig. S8). Concomitantly, proliferation of lymphocytes from immunized animals was assessed upon stimulation with uninfected salivary gland material, *Pb*WT, *Pb*Vac and *Pf* sporozoites. Our data showed that lymphocytes from either *Pb*WT-immunized or *Pb*Vac-immunized rabbits significantly responded to both *Pb*WT and *Pb*Vac sporozoite stimuli, whereas stimulation with non-infected salivary gland material induced only basal levels of thymidine incorporation (Fig. 4e and Fig. S8). As expected, stimulations with sporozoites consistently induced more marked lymphocyte proliferation than peptide-based stimulation. Lymphocytes from either group of immunized animals also incorporated significantly more ^3^H-thymidine than those of mock-immunized animals in response to a stimulus with *Pf* sporozoites, indicating not only that immunization with *Pb*Vac elicits a strong cellular response against *Pf* but also that this response occurs likewise upon immunization with *Pb*WT (Fig. 4e). These results are in complete agreement with our flow cytometry investigation of cell proliferation in the presence of the different sporozoite stimuli (Fig. S9 A-C). Furthermore, our results show that the cellular immune responses observed in *Pb*WT-immunized and *Pb*Vac-immunized animals upon sporozoite stimulation contained a major CD4^+^ T cell component (Fig. S9C). Finally, augmented lymphocyte proliferation capacity is concomitant with increased IFNɣ production, as our results show that the amount of IFNɣ produced upon stimulation of cells from sporozoite-immunized animals, but not from mock-immunized animals, with any of the sporozoite stimuli employed, was significantly enhanced compared to that of cells stimulated with non-infected salivary gland material (Fig. 4f). Overall, these data indicate not only the existence of cross-species cellular responses between *Pb* and *Pf* but also that the *Pf*CS protein engineered on the *Pb* background contributes to the cellular, and mediates the humoral, immune responses observed upon immunization with the *Pb*Vac parasite.

### Functional capacity of PbVac-induced humoral immune responses

**Fig. 5:**
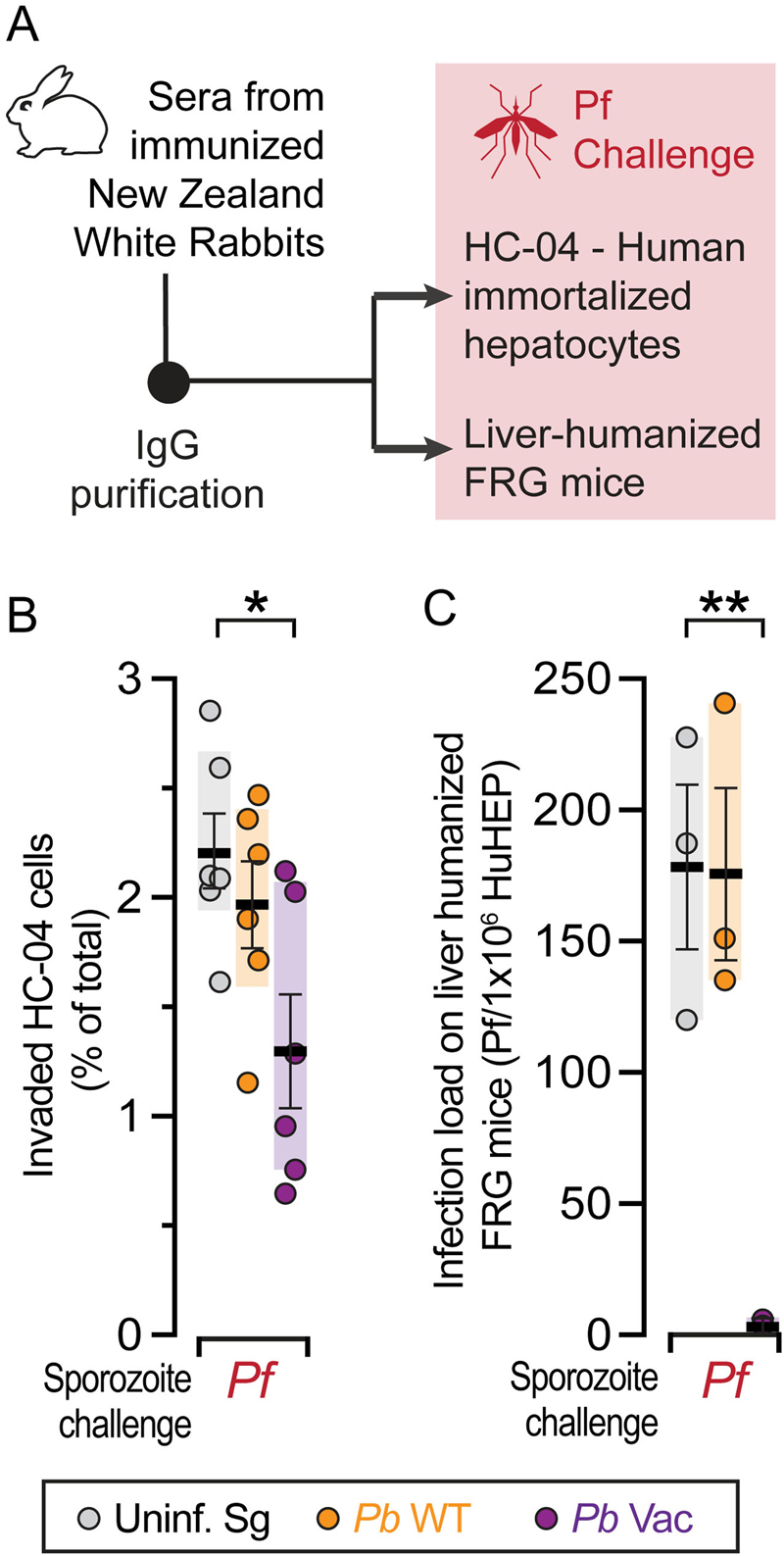
*Pb*Vac-mediated protection against *Pf* challenge. **a** Diagram of the experimental protocol. **b** *Pf* sporozoite infection of HC-04 human immortalized hepatocyte cultures incubated with purified IgG from mock- (grey), *Pb*WT- (yellow) or *Pb*Vac (purple)-immunized rabbits. **c** qRT-PCR quantification of *Pf* hepatic infection inhibition in liver-humanized FRG mice following passive transfer of purified IgGs from mock (grey)-, *Pb*WT (orange)- or *Pb*Vac (purple)-immunized rabbits and challenge by the bites of 20 *Pf*-infected mosquitoes (*n* = 3 mice per group). Measurements were taken from distinct samples. The boxes correspond to the 25th and 75th percentiles in B), and the minimum and maximum data range in C); the lines and bars indicate mean of infection and standard error of the mean, respectively; *p < 0.05; **p < 0.01, as determined by one-way ANOVA, corrected with Dunnett’s multiple comparisons test

We next evaluated the ability of the observed immune responses to inhibit infection by *Pf*. Since the protective capacity of the cellular immune responses cannot be directly assessed because the rabbit model employed is not susceptible to *Pf* infection, we focused our analysis on the functionality of the humoral immune responses elicited upon immunization. To this end, we performed both in vitro inhibition assays and in vivo *Pf* challenge assays of liver-humanized FRG mice employing IgGs isolated from the serum of mock-, *Pb*WT-immunized and *Pb*Vac-immunized rabbits (Fig. 5a). Incubation of sporozoites with ~0.2 mg/ml post *Pb*Vac-immunization IgGs led to a ~40% decrease in *Pf* infection of HC04 cells compared with IgGs from mock-immunized animals (Fig. 5b). This effect is more pronounced than the impairment observed on *Pf* sporozoite traversal following incubation with 10 mg/ml total IgGs from CPS-immunized volunteers.^33^ Finally, 10 mg total IgGs purified from the serum of mock-, *Pb*WT-immunized and *Pb*Vac-immunized rabbits were injected into liver-humanized FRG mice. Twenty-four hours after IgG passive transfer, these mice were challenged by an equal number of *Pf*-infected mosquito bites. The *Pf* load in the chimeric livers of humanized FRG mice was assessed five days later by qRT-PCR. The results show that passive transfer of 10 mg IgGs from *Pb*Vac-immunized rabbits, but not from *Pb*WT-immunized or mock-immunized animals, conveyed near complete protection against a subsequent *Pf* hepatic infection (Fig. 5c). This decrease in liver parasite load is more marked than that observed under similar experimental conditions following passive transfer of 10 mg of IgGs from CPS-immunized volunteers, which is equivalent to the typical IgG concentration in human plasma.^33^ Collectively, these data show that immunization with *Pb*Vac elicits the production of high titers of functional antibodies, mostly targeting the *Pf*CS antigen, capable of preventing a subsequent infection by human-infective parasites.

## Discussion

Despite being known for more than 100 years that attenuated pathogens can induce protective immunity,^24^ the present study introduces for the first time a practical approach to developing a human vaccine based on the concept of employing an avirulent non-human *Plasmodium* parasite as a versatile platform of WSp-based antigen delivery for vaccination against human malaria. Although transgenic rodent malaria parasites where endogenous antigens were replaced by those of their human-infective counterparts have previously been described, they served exclusively as tools for assessment of functional immunogenicity of malaria vaccines rather than as antigen delivery platforms.^32,34,35^ We now propose to use *Pb* as a safe, genetically modifiable vaccination platform that can be engineered to express multiple antigens of human malaria parasites capable of inducing functional immune responses against different *Plasmodium* species or various stages of the parasite’s life cycle.

We generated a new transgenic *Pb* parasite, *Pb*Vac, capable of expressing and delivering the *Pf* immunodominant protective antigen, *Pf*CS.^17,36^ In a pre-clinical proof-of-principle study, we show that immunisation with *Pb*Vac is effective against *Pf* through a combination of cellular immune responses and antibody-based responses. Consistent with the distribution of in silico-predicted epitopes shared between the *Pf* and *Pb* proteomes, the induction of T cell-mediated immunity by *Pb*Vac appears to be largely *Pf*CS-independent and arise from the vaccine’s *Pb* background. This is in agreement with a recent study, in which peripheral blood T cell responses to CS peptides were not detected following CPS immunization of rhesus monkeys with *P. knowlesi*,^37^ as well as with previous literature on cross-species immune responses in WSp vaccination. In fact, the first report of pre-erythrocytic cross-species protection between *Plasmodium* species dates back to 1969, when mice immunized by injection of X-irradiated *Pb* sporozoites were shown to be protected against a challenge with viable sporozoites of *P. vinckei*.^38^ Protection was also observed when *Pb*-immunized animals were challenged with rodent *P. chabaudi* sporozoites and vice versa.^39^ In other studies, mice immunized by irradiated or genetically attenuated (*p36p*^−^) *Pb* sporozoites were shown to provide partial protection against challenge by *P. yoelii* (*Py*) sporozoites,^40^ and 100% sterilizing cross-species protection was observed when mice were immunized with a late liver stage-arresting genetically attenuated *Py* parasite (*Py-abb/f*) and challenged with *Pb* sporozoites.^11^ Sedegah et al.^41^ reported T cell-mediated protection of mice immunized with attenuated *Pb* or *Py* sporozoites against heterologous challenge with *Py* or *Pb*, respectively. The requirement for T cells to provide protection against heterologous parasites was consistent with the previous observation that a *Py*CS CD8^+^ T cell clone was protective against challenge with the heterologous *Pb* sporozoites.^42^ Most relevant in the context of the present study, immunization of mice with *Pf* sporozoites protected the animals from infection with *Pb*.^43^ Protection was proposed to be mediated, at least in part, by antibody cross-reaction between antigens other than *Pf*CS and *Pb*CS, in agreement with the high genetic sequence similarity between the two parasite species.^44^ However, it should be noted that several examples of cross-species protective immune responses induced by parasite components other than CS following immunization with irradiated *Plasmodium* sporozoites are available in the literature.^45,46^ Such protective antigens may include, for instance, cell-traversal protein for ookinetes and sporozoites (CelTOS),^47^ a protein that is highly conserved among the *Plasmodium* species. Indeed, immunization of mice with *Pf*CelTOS has been shown to elicit cross-species protection against a heterologous challenge with *Pb*.^48^ In humans, CS-specific cellular immune responses were described in RAS-immunized volunteers as determined by the proliferation of peripheral blood mononuclear cells (PBMCs) upon in vitro stimulation with recombinant *Pf* CS.^49,50^ Nevertheless, the reports of human immunization with *Pf* sporozoites available so far do not show a correlation between long-term protection afforded by WSp vaccines and CS-targeted cellular immune responses, and correlations identified between long-term protection and CS-targeted antibody responses^20,21^ have been interpreted as biomarkers of vaccine take rather than mechanistic correlates. Thus, it is thought that long-term protection induced by WSp is complex and likely mediated by multiple liver stage antigens, whose identification has been a matter of previous research.^47,51^

Although protection by WSp immunisation may result from the combined activities of induced T cells and antibodies against a variety of antigens [reviewed in^18^], the presentation of a key *Pf* antigen by the *Pb* vaccination platform may provide an additional means of inducing targeted and effective immune responses. Accordingly, our results show that immunization with *Pb*Vac elicits a potent *Pf*CS antibody response, capable of functionally inhibiting infection of hepatocytes by *Pf* sporozoites. Humoral responses induced by immunization with *Pb*WT lack inhibitory activity against *Pf* sporozoites, indicating a pivotal role of the *Pf*CS protein in the antibody-based protection conferred by vaccination with *Pb*Vac. Unlike subunit approaches, a major advantage of the expression of *Pf* antigens in *Pb* is that the latter is more likely to generate full length *Pf* proteins that are correctly folded and post-translationally modified, inducing a greater array of inhibitory antibody responses against *Pf* sporozoites. Our findings are in agreement with the fact that RAS immunization of human volunteers leads to the production of high titers of antibodies against the CS protein,^49,52^ which parallel the serum inhibitory activity of sporozoite invasion of hepatoma cells in vitro.^52^ Moreover, antibodies against the immunodominant B-cell epitope of *Pf*CS were also shown to inhibit sporozoite infection in vitro^53,54^ and in vivo.^55^ Our results further indicate that the *Pf*CS component of the *Pb*Vac parasite may induce not only humoral responses but also contribute to the overall cellular responses observed after immunisation. In fact, CS-specific cellular immune responses have been described in RAS-immunized volunteers as determined by the proliferation of peripheral blood mononuclear cells upon in vitro stimulation with recombinant *Pf*CS.^49,50^ Additionally, an epitope mapping to the 5′ repeat region of *Pf*CS was identified in T cell lines and clones obtained from a sporozoite-immunized human volunteer,^56^ and another *Pf*CS epitope was shown to be recognized by human cytolytic class II-restricted CD4^+^ T cells.^57^

It is clear that WSp immunization offers several benefits over subunit vaccines, including the presentation of a wider range of antigens, correctly folded and optimally delivered to their target location. However, the success of *Pf*-based WSp vaccination depends on the strict absence of breakthrough episodes, a concern that is eliminated by *Pb*-based WSp antigen delivery systems. Since *Pb* develops into maturing liver schizonts in human hepatocytes, immunisation with *Pb* WSp vaccines is likely to result in increased antigen exposure relative to early-arresting *Pf*-based variants such as RAS or early-arresting GAP. We further observed that *Pb*Vac sporozoites are 20–50 times more infectious than *Pf*, likely increasing the effective dose of vaccination, and potentially inducing robust immune responses with relatively few immunizing parasites. Moreover, *Pb* is highly amenable to genetic manipulation, and several neutral loci have already been identified in its genome. This raises the possibility of introducing multiple antigens in the *Pb* platform, including those from different human-infective *Plasmodium* spp., as well as blood-stage or transmission-blocking antigens, placed under the control of a strict pre-erythrocytic stage promoter.

The manufacturing of a *Pb*Vac vaccine suitable for future human use can be envisaged to employ parenteral injection of sporozoites obtained from mosquitoes that fed on *Pb*Vac-infected, specific-pathogen free (SPF) rodents, previously infected with a master cell bank of *Pb*Vac parasites that is fully certified as free of human pathogens or other microbiological contaminants. A single SPF rat can be used to infect more than one thousand mosquitoes, potentially generating hundreds of vaccine doses. Importantly, the production of a *Pb*-based vaccine can be achieved in the absence of high-containment mosquito infection and handling facilities or, possibly, in vitro, as suggested by previous proof-of-principle studies.^58,59^

The data presented here employing the *Pb*Vac parasite provides the proof-of-concept that immunisation with GM rodent malaria parasites can potentially be used to protect against human malaria. Given the limitations of available animal models to predict the protective efficacy of such a vaccination approach, this can only be fully ascertained in clinical trials performed upon addressing all relevant safety and regulatory issues.

## Materials and methods

### Animal experimental procedures

Male C57BL/6 and Balb/cByJ mice, aged six to eight weeks, as well as NZW rabbits, aged four to six weeks, were purchased from Charles River and housed in the animal facilities of Instituto de Medicina Molecular, Faculdade de Medicina, Universidade de Lisboa, Portugal (iMM Lisboa). Experimental procedures were performed according to the Federation of European Laboratory Animal Science Associations (FELASA) guidelines and iMM Lisboa regulations. Blood-humanized NSG mice were produced and housed at the AAALAC-accredited GlaxoSmithKline Laboratory Animal Science facility in Tres Cantos (Madrid, Spain). All the experiments were approved by the GlaxoSmithKline Diseases of the Developing World Group Ethical Committee and complied with Spanish and European Union legislation on animal research and GlaxoSmithKline policy on the care and use of animals. Liver humanized FRG mice were produced by Yecuris (Tualatin, Oregon USA), and housed in the animal facilities of the Faculty of Medicine and Health Sciences of the Ghent University or at iMM Lisboa. The experimental protocol for *Pf* or *Pb* infection of these mice was approved by the animal ethics committees of the Faculty of Medicine and Health Sciences of the Ghent University and of iMM Lisboa (DGV-AWB-2015-09-MP-Malaria). All facilities were kept under a 12 h light/dark period at a temperature of 22 ± 2 °C and 40–70% relative humidity. Filtered tap water and a γ-irradiated pelleted diet were provided ad libitum. In experiments involving blood-stage infections, animals were euthanized at the first behavioral signs of onset of experimental cerebral malaria (ECM), and this was considered the experimental endpoint, with all efforts made to minimize animal suffering.

### *P. berghei* and *P. falciparum* reference parasite lines

The following reference lines of the ANKA strain of *Pb* were used: line cl15cy1, line 676m1cl1 (*Pb*GFP-Luccon; see RMgm-29 in www.Pberghei.eu) and line 1596cl1 (GIMO_*Pb*ANKA_ mother line; see RMgm-687 in in www.Pberghei.eu). *Pb*GFP-Luccon expresses a fusion protein of GFP and luciferase from the eef1a promoter^60^ and the GIMO-mutant contains the hdhfr::yfcu positive–negative selection marker in the silent 230p locus.^30^ For *Pf* experiments, *Pf* NF54 asexual and sexual blood stages were cultured in a semi-automated culture system. Sporozoites were obtained by dissection of salivary glands from infected female *Anopheles stephensi* mosquitoes, reared at iMM-Lisboa or at the Radboud University (Nijmegen, Netherlands), and which had previously fed on gametocyte-carrying infected mice for *Pb* infections or cultured gametocytes through standard membrane feeding for *Pf* infections. Mosquito salivary glands were kept on ice in D-MEM culture medium and homogenized with a grinder to release the sporozoites, which were subsequently counted on a Neubauer chamber.

### Generation and genotyping of transgenic *P. berghei* parasite, *Pb*Vac

A transgenic *Pb* parasite line containing a *Pfcs* expression cassette in the neutral *230p* locus was generated using the ‘gene insertion/marker out’ (GIMO) technology as previously described.^30,61^ The *Pfcs* expression cassette was introduced into the neutral *230p* locus of the GIMO mother line 1596cl1,^30,61^ using construct pL1988 (Fig. S3A). The pL1988 construct contains the *Pfcs* coding sequence (CDS) under the control of the *Pbuis4* 5′ and 3′ UTR regulatory sequences flanked by the 5′ and 3′ targeting sequences for the *230p* locus. This construct integrates by double crossover homologous recombination and replaces the positive–negative selectable marker (SM) (human *dihydrofolate* reductase:: yeast *cytosine deaminase* and *uridyl phosphoribosyl transferase* (hdhfr::y*fcu*)) cassette in the GIMO mother line 1596cl1 with the *Pfcs* expression cassette. The expression cassette contains the *Pfcs* CDS, which was amplified by PCR from *Pf* NF54 genomic DNA.^62^ The CDS is flanked by the 5′ and 3′ promoter and transcription terminator sequences of *Pb UIS4*, which were amplified from *Pb* ANKA WT genomic DNA. The coding sequence of the *Pfcs* gene was confirmed by sequencing. The construct pL1988 was linearized by digestion with by SacII before introduction into parasites of the GIMO mother line 1596cl1 using standard methods of GIMO transfection.^30^ Transfected parasites were selected in mice by applying negative selection by providing 5-fluorocytosine (5-FC) in the drinking water of mice. Negative selection results in selection of chimeric parasites where the h*dhfr*::y*fcu* SM in the *230p* locus is replaced by the *Pfcs* expression cassette (Fig. S3A). Selected transgenic parasites (line 2266) were cloned by the method of limiting dilution. Clone line 2266cl1 (*Pb*ANKA-PfCSP_*Pb*uis4_) was selected for further analysis. Correct integration of the construct into the genome of transgenic parasites was analysed by diagnostic PCR analysis of gDNA and Southern analysis of pulsed field gel (PFG)-separated chromosomes (Fig. S3B,C). Primer sequences are listed in Supplementary Tables S3 and S4.

### In vitro infection of human and mouse hepatoma cell lines

Human (Huh7, HepG2, and HC-04) and mouse (Hepa1-6) hepatoma or immortalized hepatocyte cell lines were cultured in RPMI medium supplemented with fetal bovine serum (FBS), 50 µg/mL Penicillin/Streptomycin, 2 mM Glutamine 0.1 mM non-essential amino acids (Gibco) at 37 °C with 5% CO_2_. Cells were infected 24 h after seeding, by adding 5 × 10^4^ freshly dissected sporozoites in supplemented RPMI medium containing Fungizone (1 µg/mL, Gibco), followed by a 5-min centrifugation at 3000 rpm. The number of infected hepatocytes was assessed by staining for *Plasmodium* Hsp-70 (mAb 2E6) and indirect immuno-fluorescence analysis, as previously described.^63^

### In vitro infection of mouse and rabbit PH

Mouse PH were isolated from livers of adult C57BL/6 male mice following an adaptation of a previously described perfusion method.^64^ Briefly, mouse livers were initially perfused with 30–40 mL of liver perfusion medium (LPM, Gibco) at 37 °C and a controlled flow rate of 7–9 mL/min, through a cannula inserted in the portal vein, followed by digestion with 30–40 mL of liver digest medium (LDM, Gibco). The liver was then transferred to a cell culture dish containing 10 mL of LDM, its capsule membrane removed and shaken to release loose cells. The cell suspension was then serially passed through 100 and 70 µm cell strainers, washed twice with 30 mL 4% (v/v) FBS supplemented William’s E Medium (Gibco) at 30 g for 3 min at 20 °C and purified by layering over a 60% Percoll gradient (GE Healthcare), followed by centrifugation for 20 min at 750 g, 20 °C, with no break. Purified viable hepatocytes were counted with Trypan blue and plated on collagen-coated plates before infection with freshly dissected *Pb* sporozoites (1 × 10^5^ hepatocytes infected with 7 × 10^4^ sporozoites). An adaptation of the mouse PH isolation protocol described above was applied for isolation of rabbit PH. Briefly, animals were euthanized by injection of 150 mg/kg IV Sodium Pentobarbital (Eutasil, CEVA), exsanguinated by direct heart puncture and immediately opened to begin the process of liver perfusion. The portal vein was cannulated with a 21G needle and the inferior vena cava cute for outflow. Perfused occurred with 400–500 mL of liver perfusion medium (LPM, Gibco) at 37 °C followed by digestion with 400–500 mL of liver digest medium (LDM, Gibco) at a flow rate of 18 mL/min, controlled by a peristaltic pump. The liver was carefully removed, cut in small pieces and gently shaken to release loose cells. The cell suspension was then treated identically to mouse PH to isolate purified viable hepatocytes which were plated on collagen-coated plates and allowed to settle and attach overnight. *Pb* infection and development was assessed by immunofluorescence using mouse anti-*Plasmodium* Hsp-70 (mAb 2E6), rabbit anti-*Pb* MSP1, goat anti-*Pb* UIS4, mouse anti-*Pb* CS (mAb 3D11) and mouse anti-*Pf* CS (mAb 2A10) and appropriate secondary antibodies, as previously described.^63^

### In vitro infection of human PH

*Pb* and *Pf* infection of human PH was performed on either micropatterned co-culture (MPCC) preparations as previously described^65^ or on cultures of fresh primary human hepatocytes isolated from patients undergoing partial hepatectomy. Briefly, for micropatterned co-cultures, 1 × 10^4^ cryopreserved primary human hepatocytes (Life Technologies) were seeded on collagen-micropatterned plates softlithographically patterned with 500 µm islands together with 7 × 10^3^ 3T3-J2 murine embryonic fibroblasts. One day after seeding, 7 × 10^4^ freshly dissected *Pb* sporozoites were added to each well and subsequently, cells were fixed at various time points post infection for immunofluorescence microscopy analysis. For fresh primary human hepatocyte culture, viable hepatocytes were seeded into collagen-coated 96-well flat-bottom plates (5 × 10^4^ hepatocytes/well) in complete William’s B medium and cultured at 37 °C in an atmosphere of 5% CO_2_. Two days after seeding, a batch of 5 × 10^4^ freshly dissected *Pf* or *Pb* sporozoites were added to each well. Cells were fixed 2 and 5 days after infection for *Pb* and for *Pf*, respectively. The number of infected hepatocytes was assessed by staining for *Plasmodium* Hsp-70 (mAb 2E6) and indirect immunofluorescence analysis.

### In vivo infection of liver humanized FRG mice

Highly repopulated FRG mice were obtained from Yecuris (Tualatin, Oregon, USA), as previously described.^66^ Briefly, six-to-eight weeks old mice received an injection in the spleen of cryopreserved human hepatocytes from a single donor. The mice were then subjected to standardized NTBC (2-(2-nitro-4-trifluoromethylbenzoyl)-1,3-cyclohexanedione) withdrawal regimen. Starting eight weeks post transplantation, human albumin levels are monitored using the Bethyl Laboratory Quantitative Human Albumin ELISA Kit (Catalog #E90-134) according to the manufacturer’s protocol. Mice with albumin levels above 5 mg/mL, corresponding to <95% repopulation, were included in the study. For *Pb* infection, liver chimeric FRG humanized mice were injected respectively with 2 × 10^6^ sporozoites or uninfected mosquito salivary glands as control. Mice were euthanized 48 h post infection and parts of the liver were either preserved in RNAlater, or placed in 4% paraformaldehyde for 24 h, after which they were stored in *PB*S and further processed to paraffin blocks. For microscopy analyses of *Pb*-infected livers, liver sections were either stained with hematoxylin and eosin following standard procedures or a combination of anti-*Pb*UIS4 antibody and anti-fumarylacetoacetate hydrolase (Yecuris®) and/or by anti-hepatocyte specific antigen antibody (OCH1E5, Santa Cruz®). For *Pf* challenge, mice received an intraperitoneal injection of either 10 mg rabbit IgG purified from immunized rabbits, or *PB*S, in an adaptation of a previously established methodology.^33^ Briefly, IgG purification from 3–8 ml plasma samples was performed using Ab SpinTrap columns (GE Healthcare Life Sciences), according to the manufacturer’s instructions and purified IgGs were further concentrated to 200 µl using Amicon Ultra-0.5 Centrifugal Filter Units with Ultracel-30 membrane (Millipore) also according to the manufacturer’s instructions. Twenty-four hours after injection, animals were exposed to the bites of 20 *Pf*-infected mosquitoes for 20 min. Successful blood feeding and sporozoite presence was confirmed by mosquito dissection after the challenge experiment. Five days later, mice were euthanized and liver parasite burden (ring stage equivalent parasites (*Pf*) per 10^6^ human hepatocytes) was determined as previously described.^67^ Each liver was divided into 12 sections, of which 25 mg tissue was weighed and DNA was extracted into 100 µL elution buffer with the High Pure PCR Template Preparation Kit (Roche, Zaventem Belgium). *Pf* DNA levels were quantified using a highly sensitive qPCR assay. qPCR was also employed to assess the degree of repopulation with human hepatocytes of the chimeric livers, and to normalize the *Pf* copy numbers.

### In vivo infection of blood-chimeric humanized mice

Highly engrafted mice with human erythrocytes (hE) were obtained and infected as previously described.^29^ Briefly, NOD-*scid IL-2Rγ*^*null*^ mice (NSG) obtained from Charles River laboratories were injected i.p. daily throughout the experiment with hE. When 60–70% chimerism in peripheral blood was reached (7–10 days after initiation of injections), the mice were infected by iv injection of 1 × 10^7^ parasites obtained from infected donors or 5 × 10^5^ freshly dissected sporozoites (not shown). Parasitemia was measured during the following days by flow cytometry analysis of 2 µl of blood collected from the tail lateral vein of infected mice as previously described.^68^ Blood was rapidly transferred into 0.1 ml of saline containing 2.5 or 5 µM SYTO-16 (for *Pb*) and 10 µg/ml TER119-PE (for murine erythroid lineage), and incubated for 20 min at room temperature in the dark. Ten microliter of saline containing 0.25% (w/v) glutaraldehyde was then added to each sample and incubated for an additional 5 min for complete *Plasmodium* inactivation. The samples were then analysed on FACScalibur or LSRII flow cytometers (Becton Dickinson). Erythrocytes were gated based on side scatter and forward scatter analysis, followed by a key compensation step for SYTO-16 to accurately define the region of infected events by comparison of the effect of increasing compensation of the emission of SYTO-16 in samples from uninfected and *Plasmodium*-infected mice. Results were analyzed using either CellQuest-Pro or BD FACSDiva 5.0 (Becton Dickinson) software. Microscopic analysis of samples of peripheral blood from infected mice was simultaneously performed in blood smears stained with 10% (v/v) Giemsa in saline buffer (0.015 M NaCl, 0.001 M phosphate buffer, pH 7.0).

### In silico identification of CD8^+^ T cell epitopes in the *Pf* and *Pb* proteomes

The complete proteomes of *Pf* and *Pb*, consisting of 5548 and 5076 annotated proteins, respectively, were downloaded from PlasmoDB (v36).^69^ CD8^+^ T cell epitope prediction was performed with the package NetMHCpan (v4.0),^70^ and was based on ten alleles representative of the HLA-A and HLA-B supertypes, with allele frequencies as described in the Allele Frequency Net Database,^71^ accessed on 22 February 2018. Peptide lengths of 9, 10, and 11 residues were used to search for 9 residue-long core epitopes. Reported results are based only on strong binders, which are defined as those in the top 0.5% of affinity binding prediction scores, according to best practices.

### Data availability

The datasets generated during and/or analysed during the current study are available from the corresponding author on reasonable request.

## Electronic supplementary material

Supplemental Material

## Electronic supplementary material

**Supplementary information** accompanies the paper on the *npj Vaccines* website (10.1038/s41541-018-0068-2).
