# Supplementary material for: A Plasmodium berghei sporozoite-based vaccination platform against human malaria
Source: NPJ Vaccines. 2018 Aug 24;3:33. doi: 10.1038/s41541-018-0068-2 (PMC6109154; doi:10.1038/s41541-018-0068-2)
Supplement: Supplementary file 1 — Supplemental Material [file 41541_2018_68_MOESM1_ESM.pdf]

Supporting Information for

A *Plasmodium berghei* Sporozoite-Based Vaccination Platform Against Human Malaria

António M. Mendes, Marta Machado, Nataniel Gonçalves-Rosa, Isaie Reuling, Lander Foquet, Cláudia Marques, Ahmed M. Salman, Annie S.P. Yang, Kara A. Moser, Ankit Dwivedi, Cornelus C. Hermsen, Belén Jiménez-Díaz, Sara Viera, Jorge M. Santos, Inês Albuquerque, Sangeeta N. Bhatia, John Bial, Iñigo Angulo-Barturen, Joana C. Silva, Geert Leroux-Roels, Chris J. Janse, Shahid M. Khan, Maria M. Mota, Robert W. Sauerwein, Miguel Prudêncio*

*correspondence to: mprudencio@medicina.ulisboa.pt

SI Materials and Methods

***In vitro* culture of *P. berghei*-infected mouse and human RBC.** A magnetic cell separation procedure was used to separate mouse and human red blood cells in blood-chimeric mice. Microbead magnetic particles conjugated to anti-Ter-119 antibody were used to secure mouse erythrocytes on a separation column in the presence of a magnet. Both the positive fraction (mouse erythrocytes) and the negative fractions (human erythrocytes) were recovered. Briefly, approximately 1 mL of blood was collected by heart puncture from infected blood-chimeric mice and washed with *PB*S, resuspended in 0.25 ml of *PB*S containing 1% (v/v) FCS (Sigma) and stained with biotinylated rat anti-mouse TER-119 (BD Biosciences Pharmingen) at 10 mg/ml for 30 min at room temperature. After washing twice with separation buffer (PBS containing 0.5% (v/v) BSA and 2 mM EDTA), the cells were resuspended in 0.45 ml of this buffer and 50 mL of streptavidin conjugated BD iMag DM particles (BD Biosciences Pharmingen) and incubated for 30 min at 4 ºC.

Finally, 0.55 ml of separation buffer was added to the sample and the cellular suspension was exposed to DYNAL MPC-1 Magnetic Particle Concentrator (Dynal, Oslo, Norway) for 6 min. The quality of purification was assessed by flow cytometry as described above and purity was found to range between 87 and 95%. Both positive and negative fractions were cultured in vitro using standard conditions. Briefly, parasites were cultured using RPMI-1640 supplemented with 0.5 % Albumax and 150 µM hypoxanthine at 2 % haematocrit adjusted by the addition of either human or mouse red blood cells under an atmosphere of 90 % N2, 5 % CO2, 5 % O2 at 37 °C. At various times after incubation, small samples were collect for microscopy analysis of parasite development by Giemsa staining of cultured blood smears.

***In vivo* infection of mouse and rabbit livers.** Mice and rabbits were infected by either being exposed to infected mosquito bites or by intravenous injection of freshly dissected sporozoites. For mice infection, $1x10^4$ or $5x10^4$ freshly isolated *Pb* sporozoites were injected intravenously in the ocular plexus. For rabbit infections, sedated animals (25 mg/kg Ketamine (Imalgene 1000, Merial) + 0.25 mg/kg Dexmedetomidine (Dexdomitor, Pfizer) administered subcutaneously and reverted with 0.2 mg/kg Atipamezole (Antisedan, Zoetis), also administered subcutaneously) were exposed to the bites of ~75 infected mosquitoes or were injected intravenously in the ear vein with defined amounts of sporozoites ($1x10^5$, $5x10^5$ or $1x10^6$). Selected mouse liver lobes and small (~1 cm^3) pieces of rabbit livers were isolated from infected animals at various times post infection and fixed with 4% (v/v) paraformaldehyde. Mouse liver lobes were fixed at room temperature for 2 to 4 h, while the small pieces of rabbit livers were fixed first for 4 h at room temperature followed by overnight at 4 ºC. The fixed liver lobes or pieces were cut into 50 µm-thick

sections using a Vibratome VT 1000S (Leica). Liver sections were blocked in 2% (w/v) bovine serum albumin and 0.5% (v/v) Triton X-100 at 4 °C overnight, stained with goat anti-*Pb* UIS4, mouse anti-*Pb* CS (mAb 3D11) and mouse anti-*Pf* CS (mAb 2A10). The secondary antibodies used for detection were: Alexa Fluor 555 donkey anti-goat antibody and donkey anti-mouse conjugated to Alexa Fluor 488 (all 1:500). Cell nuclei were stained with Hoechst and F-actin with Alexa Fluor® 647 phalloidin. Stained liver sections were mounted on microscope slides with Fluoromount-G (SouthernBiotech). Images were acquired on a LSM 710 and/or a LSM 510 Meta confocal point-scanning microscope (Zeiss).

ELISA. High protein-binding capacity 96 well enzyme-linked immunosorbent assay (ELISA) plates (Nunc MaxiSorp™ flat-bottom) were coated with synthetic peptide (Sigma) based on the repeat region of the *Pf*CS protein with the amino acid sequence (NANP)4NVDPC or the repeat region of the *Pb*CS protein with the amino acid sequence (DPPPPNPN)2. The peptide was coated overnight at 4°C at a concentration of 5 µg/mL in a volume of 50 µL per well. Plates were washed three times with *PB*S containing 0.1% (v/v) Tween-20 and blocked with 200 µl *PB*S containing 0.1% (v/v) Tween-20 and 1% (w/v) BSA for 30 min at room temperature. Plates were washed one additional time and serially diluted in *PB*S containing 0.1% (v/v) Tween-20 and 1% (w/v) BSA were added and incubated at 22 °C for 2 h. After washing four times, peroxidase labeled goat anti-rabbit IgG (GE Healthcare UK) was added at a dilution of 1:2000 and incubated at 22 °C for 1 h. After washing four times, BD OptEIA™ TMB Substrate Reagent was added for development and incubated for 1 to 3 minutes at 22 °C before the stopping the reaction by addition of 50 µl Stop solution (2N H_2SO_4). The Optical density was determined using a

microplate reader (Infinite M200). To serve as a positive control and to allow comparison between samples from different assays, a standard titration curve of at least of 8 points starting a dilution of 1/20 of a pool of rabbit sera from all immunized animals was used as reference in all assays.

Sporozoite Immunofluorescence Assay (IFA). Sera collected from immunized animals were tested by immunofluorescence using air-dried methanol- fixed *Pf* sporozoites, in order to detect antibody binding to sporozoites. Briefly, purified *Pf* sporozoites (NF54 strain) were suspended in *PB*S, pH 7.4 so that 5x10^3 sporozoites could be added to each well of Thermo Scientific™ Hydrophobic Printed Well Slides using a volume of 5 µl per well. Slides were then left at room temperature overnight for air-drying, and then stored at -20 °C until used. Rabbit sera samples was serially diluted in *PB*S, starting at 1:50, and 20 µl were added to the wells. Samples predicted to be negative were only used at 1:50 and all samples were incubated at 37 °C for 45 min in a humid chamber. Slides were then washed three times with *PB*S and incubated with mouse monoclonal antibody against the *Pf*CS protein, 2A10, at a 1:2000 dilution, as a positive control for the presence of *Pf* sporozoites. Alexa fluor 488 conjugated donkey anti-mouse IgG (Molecular Probes) and Alexa fluor 568 conjugated donkey anti-rabbit IgG (Molecular Probes) were diluted to 1:500 in *PB*S and 20 µL were added to each well, followed by an incubation of 45 min at 37 °C in a humid chamber. Slides were washed three times with *PB*S and mounted with in Fluoromount-G® (SouthernBiotech 0100-01) mounting medium. Samples were analyzed on a Zeiss Axiovert 200M microscope at 400 X magnification. The endpoint titer was defined as the last serum dilution at which fluorescence intensity was higher than the negative controls provided by sera samples from animals subjected to uninfected mosquito bites.

***In vitro* cell proliferation analysis.** Spleen cells were isolated from the spleen of immunized rabbits. Briefly, rabbits were sacrificed by intravenous injection of 150 mg/kg IV Sodium Pentobarbital (Eutasil, CEVA). Blood was collected by cardiac puncture into heparinized tubes and the spleen collected into a 50 ml tube containing *PB*S with 2% (v/v) FBS on ice. Spleen cell suspensions were made by passage of the organ through a 70 µm nylon cell strainer (BD Labware) and spleen erythrocytes were lysed by 3 min incubation on ice in ACK lysing solution. After erythrocyte lysis, cells were incubated for 10 min at 37°C, in *PB*S without fetal calf serum (FCS), with 1 µM CellTrace Violet (Invitrogen Inc., USA). After this incubation, extracellular dye was neutralized by the addition of FCS at final concentration of 10% (v/v), and cells were centrifuged for 5 min, 400 g, at 4°C. $1x10^6$ purified spleen cells were resuspended in complete RPMI medium, and incubated with different stimuli for 7 days, at 37 °C, in a humidified cell culture incubator. Cells were incubated with overlapping peptides of *Pb*CS and *Pf*CS (15 amino acid-long peptides with 12 overlapping amino acids, covering the entirety of each protein) according to the annotated sequences available in PlasmoDB (PBANKA_0403200 and PF3D7_0304600, respectively) at a final concentration of 2µg/ml. For sporozoite stimulation assays, $5x10^4$ sporozoites/ml were employed. The proliferation levels were measured by the CellTrace Violet dilution, in comparison with non-stimulated cells, where dye dilution is minimal or does not occur. Alternatively, 1 mCi [^{3}H]thymidine (Amersham) was added for the last 18 h, and [^{3}H]thymidine incorporation was measured using the MicroBeta TriLux scintillation counter (PerkinElmer).

IFN-γ production. Mononuclear leucocytes were isolated from immunized rabbit's spleens by Ficoll density sedimentation. Single-cell suspensions were cultured *ex vivo* for 120 h after specific stimuli, as described above for cell proliferation assays. Cell cultured supernatants were collected for IFN-γ cytokine quantification by enzyme-linked immunosorbent assay (ELISA) according to the manufacturer's recommendations (Rabbit IFNγ Do-It-Yourself ELISA kit – Kingfisher Biotech, Saint Paul, MN, USA). The kit contains capture antibody, standard, and detection antibody. In each ELISA assay, a standard curve was generated using several dilutions of the recombinant rabbit IFN-g protein (detection range from 0.4 to 15.6 ng/ml). Briefly, Nunc MaxiSorp™ flat-bottom 96-well ELISA plate (ThermoFisher Scientific Inc., Waltham, MA, USA) was coated overnight with 2.5 μg/ml of anti-rabbit IFN-g polyclonal capture antibody. The biotinylated anti-rabbit IFN-g polyclonal detection antibody (0.1 μg/ml) was added to 96-well ELISA plates after previous incubation of specific standards and experimental samples. Next, streptavidin-horseradish peroxidase conjugated complex (BD Biosciences, Franklin Lakes, NJ, USA) was added followed by 3,3',5,5' tetramethylbenzidine (TMB) substrate reagent (BD Biosciences, Franklin Lakes, NJ, USA) and stop solution (2N H_2SO_4). Absorbance was measured using an Infinite® 200 PRO microplate reader (Tecan, Männedorf, Switzerland) at 450 nm and cytokine IFNγ levels were calculated. Data were expressed as ng/ml.

Inhibition of Sporozoite Invasion Assay. To assess the functional capacity of IgG from immunized animals to inhibit cell traversal by sporozoites, HC-04 human immortalized cells (MRA-975) were cultured with F-12 Nutrient Mixture (GIBCO) and Minimal Essential Medium (GIBCO) supplemented with 10% FBS (GIBCO) and 1% (v/v)

penicillin/streptomycin (GIBCO), at 37 °C in an atmosphere of 5% CO2 and infected by *Pf* sporozoites. Sporozoites were pre-incubated with 0.2 mg/ml IgG for 30 min on ice and $5x10^4$ sporozoites were added to 96-well plates containing monolayers of $5x10^4$ HC-04 hepatocytes in triplicates. Plates were then centrifuged at 3,000 RPM for 10 min at RT with a low brake (Eppendorf Centrifuge 5810 R) and incubated for 24 h at 37 °C in 5% CO2. After incubation, wells were gently washed three times with *PB*S, trypsinized with 0.05% Trypsin-EDTA (GIBCO) for 5 min at RT, taken up in 10% (v/v) FBS in *PB*S, and centrifuged at 3,600 RPM for 5 min at RT (Eppendorf Centrifuge 5415 D). Cells were re-suspended in 1% PFA in *PB*S and stored at 4 °C in the dark until analysis by flow cytometry on an ADP Cyan flow cytometer (Beckman Coulter) following staining with an anti-Hsp70 antibody. Sporozoite invasion was corrected for background and the percentage of inhibition of invasion was calculated relative to that of control samples.

In silico **analysis to identify overlapping $CD8^+$ T cell epitopes between *Pf* and *Pb*.**

Epitopes were predicted in the proteomes of *Pf* and *Pb* as described in Methods. Epitope prediction by NetMHCpan (v4.0) [70] was conducted on proteins with identifiers as listed in PlasmoDB (v36) [69], and therefore the predicted epitopes were automatically assigned the identifier of the protein in which they were found. Then, for each *Plasmodium* species, the output of NetMHCpan (v4.0) [70] was parsed to obtain all unique epitopes predicted across all HLA supertypes and across all epitope lengths. These two sets of unique epitopes, one in each species, were then compared, to identify those common to both species. The proteins containing shared epitopes were compared to the file of orthologs obtained from Orthomcl.org (v5) (orthomcl.org/common/downloads/release-5/pairs/orthologs.txt.gz), to identify pairs of *Pb* and *Pf* proteins belonging to the same orthologous group.

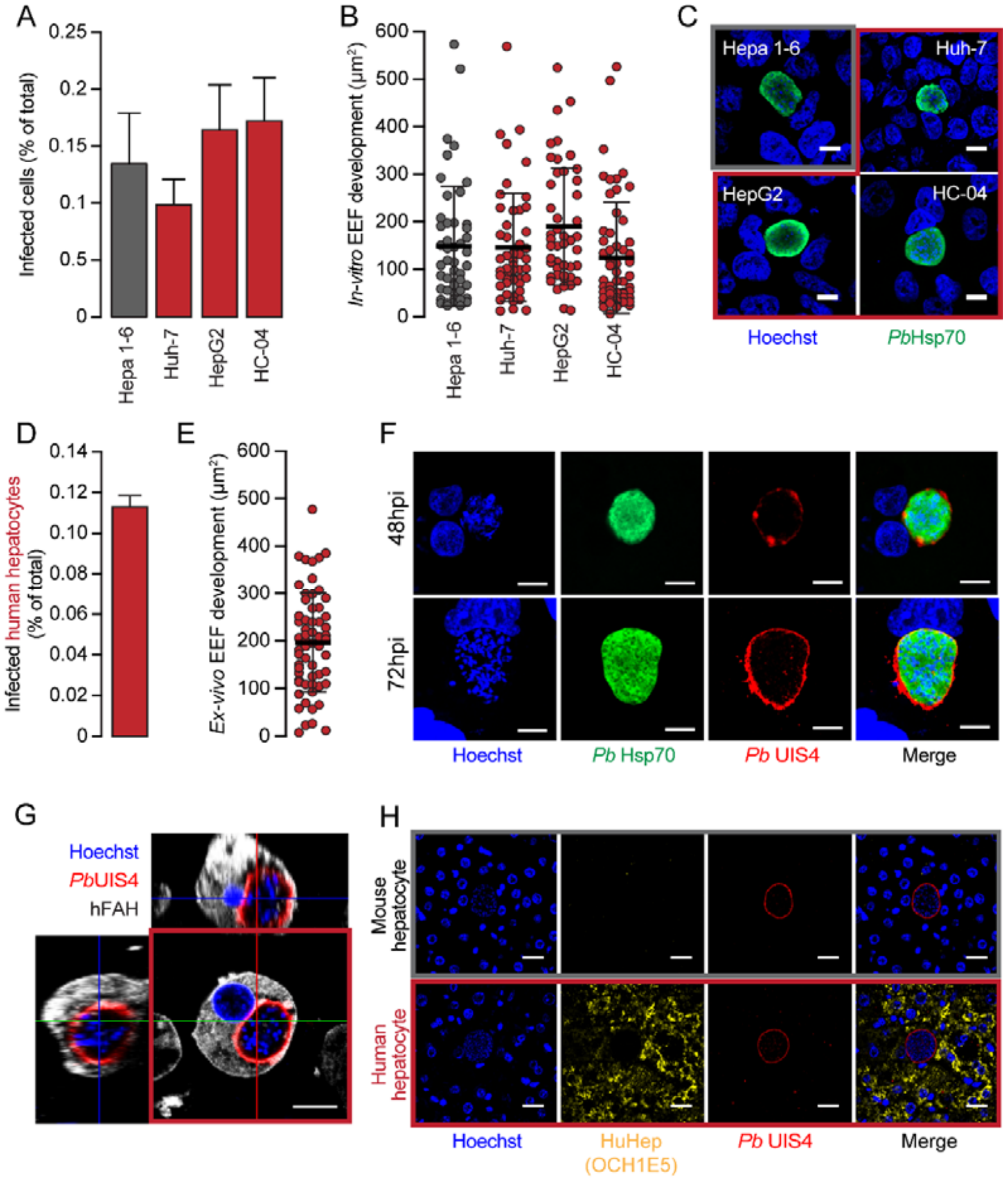

Fig. S1. *P. berghei* sporozoite invasion and development in murine and human liver cells. **(A,B)** Infection (A) and 48 hpi development (B) of *Pb* parasites in murine (grey) and human (red) hepatoma cell lines by immunofluorescence microscopy; **(C)** Representative images of *Pb* parasites 48 hpi in murine (grey square) and human (red squares) hepatoma cell lines. **(D,E)** *Pb ex vivo* infectivity (D) and development (E) in micropatterned human primary hepatocytes/fibroblast co-cultures; **(F)** Representative immunofluorescence microscopy images of *Pb* EEFs 48 (upper panels) and 72 (lower panels) hpi of micropatterned human primary hepatocytes/fibroblast co-cultures; **(G,H)** Images and projections of *Pb* parasites developing 48 hpi inside human hepatocytes of liver humanized FRG mice. Note that the *Pb* parasite, identified by the specific stain of the *Pb*UIS4 protein (red) is clearly within a human hepatocyte, as identified by fumarylacetoacetate hydrolase-stain (white, in G) or by anti-hepatocyte specific antigen antibody (OCH1E5, yellow, in h). Scale bars correspond to 10 µm.

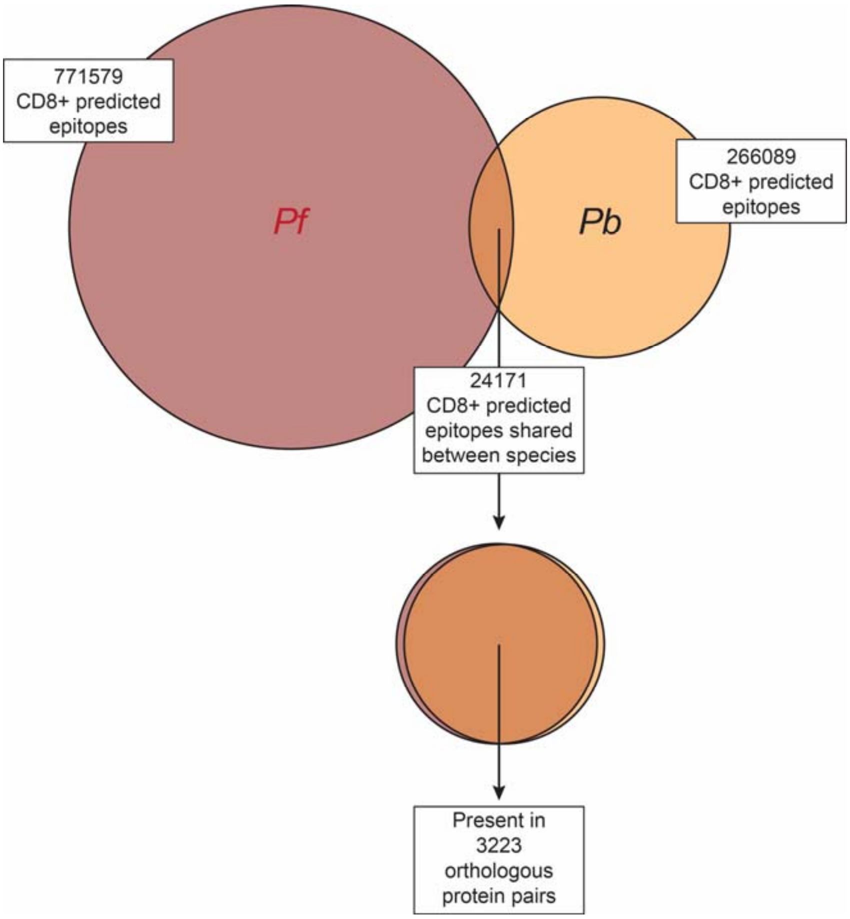

Fig. S2. Distribution of shared strong binding epitopes between the *Pf* and *Pf* proteome.

24171 *in silico*-predicted epitopes are shared between species (Table S1). These are encoded in 3371 *Pf* proteins and 3332 *Pb* proteins, of which 3223 are orthologous pairs in the two species. *Pf* proteins containing shared epitopes include several antigens expressed during pre-erythrocytic stages, including SLARP (PF3D7_1147000), SIAP1 (PF3D7_0408600), LISP1 (PF3D7_1418100) and MB2 (PF3D7_05166). Notably missing from the *Pf* proteins containing shared epitopes is the CS protein.

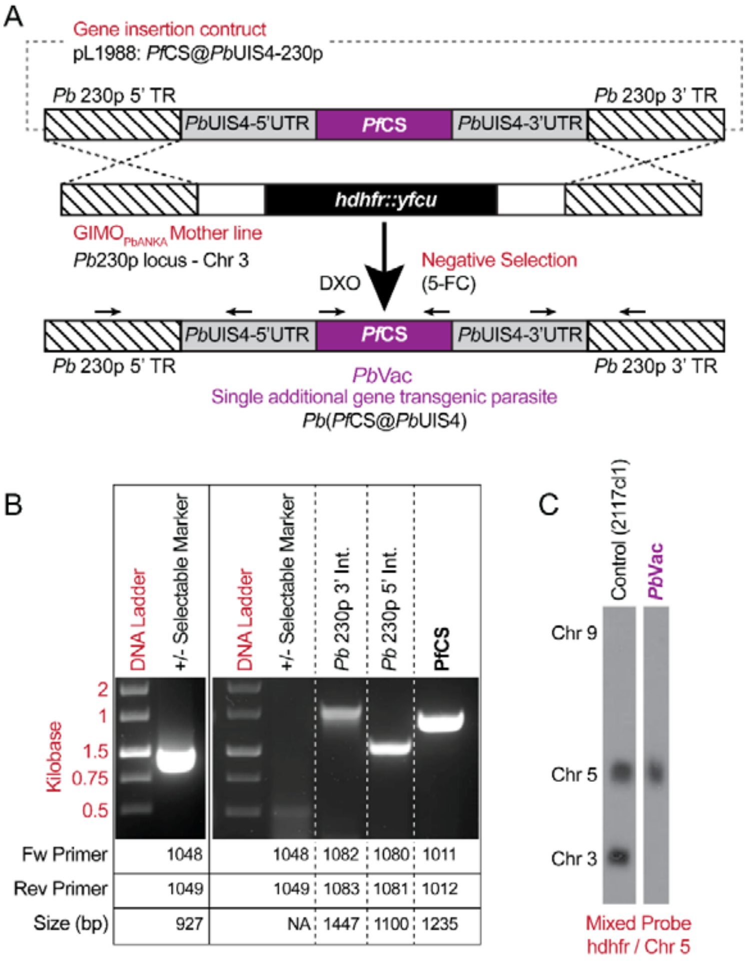

Fig. S3. Generation and genotyping of the transgenic *P. berghei* line. **(A)** Schematic representation of the transgenic line *Pb*Vac line (*Pb*(*Pf*CS@UIS4) where the GIMO insertion-construct (pL1988) replaces the selectable marker (SM; h*dhfr*::*yfcu*) in the GIMO *Pb*ANKA mother line with the *Pf*CS coding sequence (CDS) after negative selection using 5-fluorocytosine (5-FC). Construct pL1988 integrates by double cross-over homologous recombination (DXO) using 5' and 3' targeting sequences (TR) for the neutral *230p* locus, resulting in the introduction of the *Pf*CS CDS under the control of the *Pb*UIS4 gene promoter (5'-UTR) and *Pbuis4* transcriptional terminator sequence (3'- UTR) and removal of the SM. Black arrows: location of primers used for diagnostic PCR; **(B)** Genotype analysis by diagnostic PCR analysis of the cloned parasite *Pb*Vac line confirms correct integration of the *Pf*CS expression cassette in the neutral *230p* locus. Correct integration is shown by the absence of the h*dhfr*::*yfcu* SM, the presence of the *Pf*CS CDS, and the correct integration of the construct into the genome both at the 5' and 3'regions (5'int. and 3'int.). Primers sequences used are shown in Supplementary Tables 1 and 2, while the expected PCR product sizes and the primer numbers are listed in the table below the PCR analysis; **(C)** Genotype analysis by Southern analysis of pulsed-field gel electrophoresis (PFGE) separated chromosomes (chr.). The correct integration of the *Pf*CS expression construct (pL1988) into the *230p* locus of the GIMO *Pb*ANKA mother line was confirmed by showing the removal of the h*dhfr*::*yfcu* selectable marker (SM) cassette from chr. 3 in the cloned parasite *Pb*Vac line. The southern blot is hybridized with a mixture of two probes: one recognizing h*dhfr* and a control probe recognizing chr. 5. As an additional control (ctrl), parasite line 2117 cl1 is used with the h*dhfr*::*yfcu* SM integrated into chr. 3.

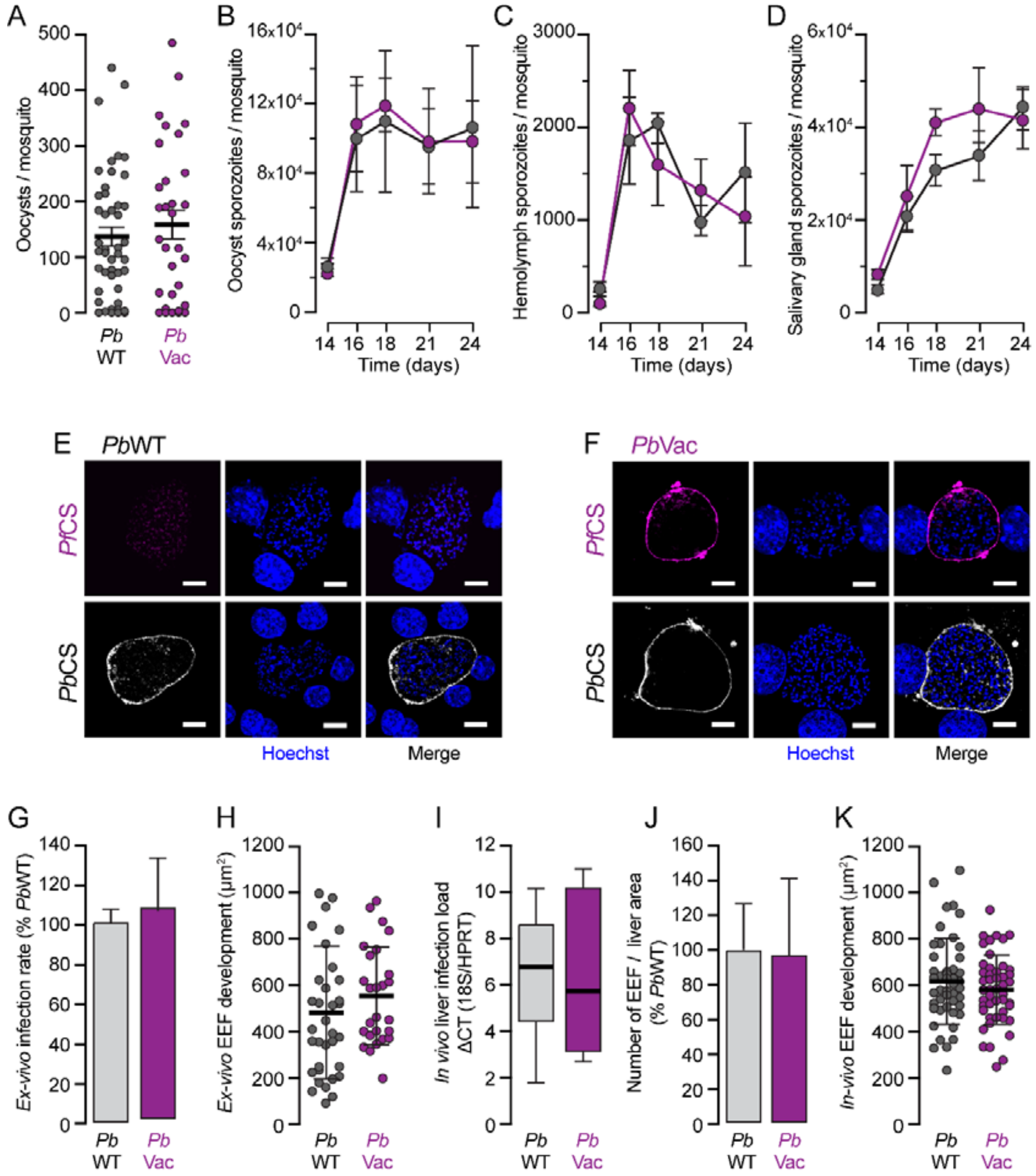

Fig. S4. *Pb*Vac and wild-type *P. berghei* sporogonic and pre-erythrocytic development. **(A,B,C and D)** Oocyst (A), oocyst sporozoite (B) hemolymph sporozoite (C) and salivary gland sporozoite (D) numbers in WT *Pb*- and *Pb*Vac-infected mosquitoes; **(E,F)** Representative immunofluorescence microscopy images of *Pf*CS (purple) and *Pb*CS (white) expressed by WT *Pb* (E) and *Pb*Vac (F) parasites developing in *ex vivo* cultures of mouse primary hepatocytes;**(G,H)** Comparative infectivity (G) and parasite development (H) of WT *Pb* and *Pb*Vac parasites developing in *ex vivo* cultures of mouse primary hepatocytes; **(I,J,K)** Comparative *in vivo* infectivity and development of WT *Pb* and *Pb*Vac parasites as determined by qRT-PCR analysis of mouse livers (I) quantification of the number of parasites developing per liver area (J), and development of hepatic EEFs (K).

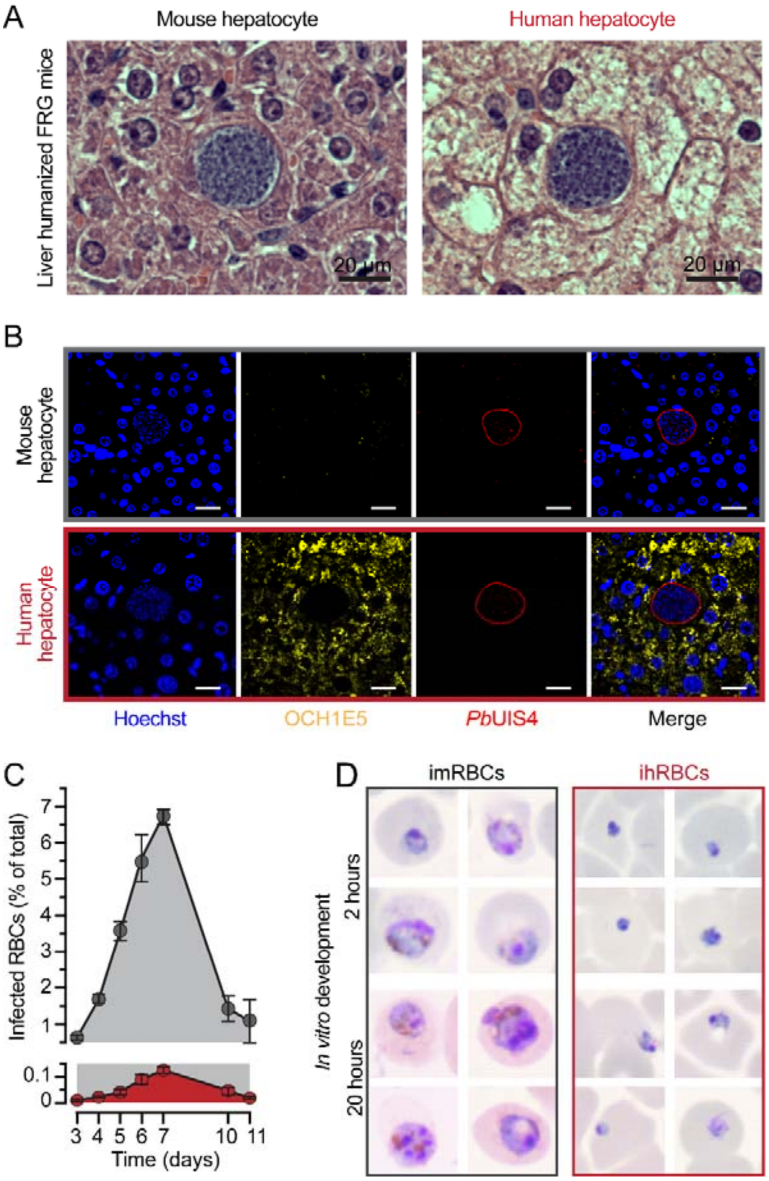

Fig. S5. *Pb*Vac and wild-type *P. berghei* infection and development in human hepatocytes and RBCs.

(A,B) Representative images of *Pb*Vac parasites 48 hpi of mouse and human hepatocytes in liver humanized FRG mice, identified by differential eosin staining (A) and by immunofluorescence staining with the anti-human hepatocyte antibody OCH1E5 (yellow, in B); **(C)** Relative proportion of *Pb*Vac-infected mouse and human RBCs following infection of blood humanized NSG mice; **(D)** Representative images of *Pb*Vac parasite forms observed within imRBCs and ihRBCs after 2 and 20 hours of *in vitro* culture, showing the inability of *Pb*Vac to develop within ihRBCs.

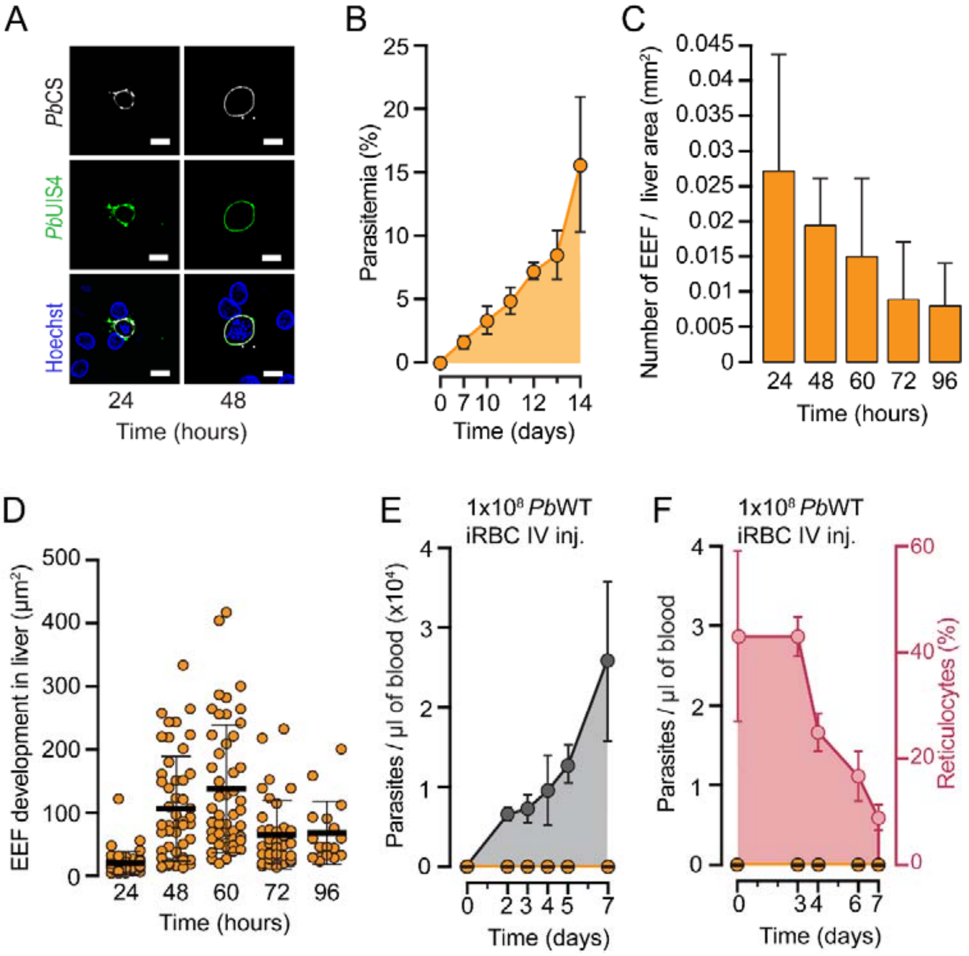

Fig. S6. *Pb* infection and development in New Zealand White Rabbits.
(A) Representative immunofluorescence microscopy images of *Pb* parasites developing *ex vivo* within rabbit primary hepatocytes at 24 and 48 hours post infection; **(B)** Dynamics of *Pb* blood stage development in mice infected by intravenous inoculation with merosomes formed in a *Pb* sporozoite-infected *ex-vivo* culture of rabbit primary hepatocytes. **(C, D)** Relative number of infected hepatocytes (C) and overall parasite development (D) observed in livers of rabbits inoculated with *Pb*WT sporozoites.; **(E)** Parasitemia in the peripheral blood of rabbits (orange) or control mice (grey) inoculated with *Pb*WT iRBC; **(F)** Parasitemia in the peripheral blood of rabbits (orange) under conditions of enhanced reticulocytemia (red).

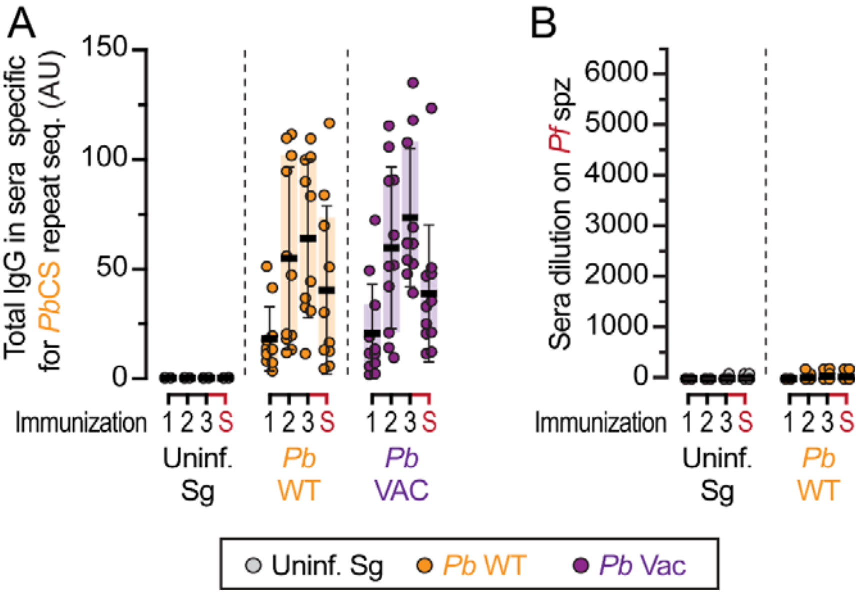

Fig. S7. Immunization with *Pb*Vac parasites elicits robust humoral responses. **(A)** Total serum IgG titers against the *Pb*CS repeat sequence after 1, 2 and 3 immunizations or at the time of animal sacrifice (S) (mock immunized- grey, *Pb*WT immunized- orange, *Pb*Vac - purple); **(B)** Binding capacity to *Pf* sporozoites of serum samples collected after 1, 2 and 3 mock (grey) or *Pb*WT immunizations, or at the time of animal sacrifice (S); The boxes correspond to the 25th and 75th percentiles; the line and bars indicate mean of infection and standard error of the mean, respectively.

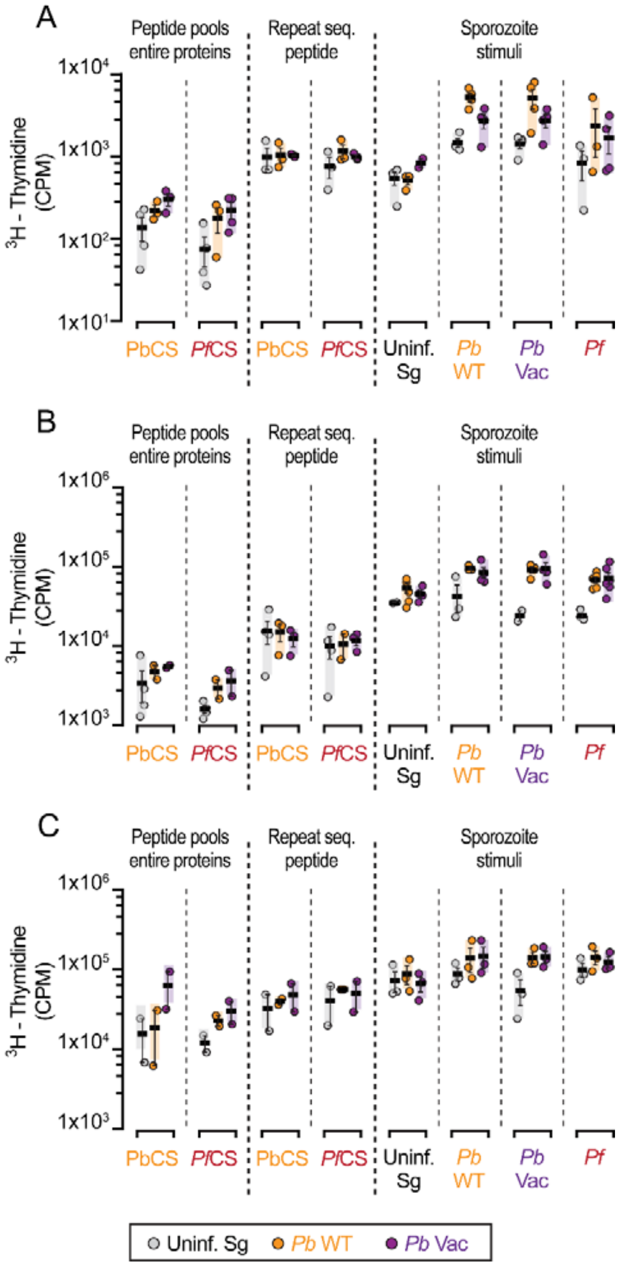

Fig. S8. Comparative analysis of cellular immune responses induced upon immunization with *Pb*Vac sporozoites.

(A, B, and C) Individual biological replicates for experiments assessing proliferation of lymphocytes from immunized rabbits as measured by ^{3}H-thymidine incorporation. Concomitant stimulation of spleen cells with either peptide pools spanning the entire *Pb*CS or *Pf*CS proteins, or peptides representing to the repeat sequences of *Pb*CS or *Pf*CS proteins, or extracts of uninfected mosquito salivary gland material and *Pb*WT, *Pb*Vac or *Pf* sporozoites allows for the direct comparison between intensity of response and consistency across biological replicates; The boxes correspond to the minimum and maximum data range; the line and bars indicate mean of infection and standard error of the mean, respectively.

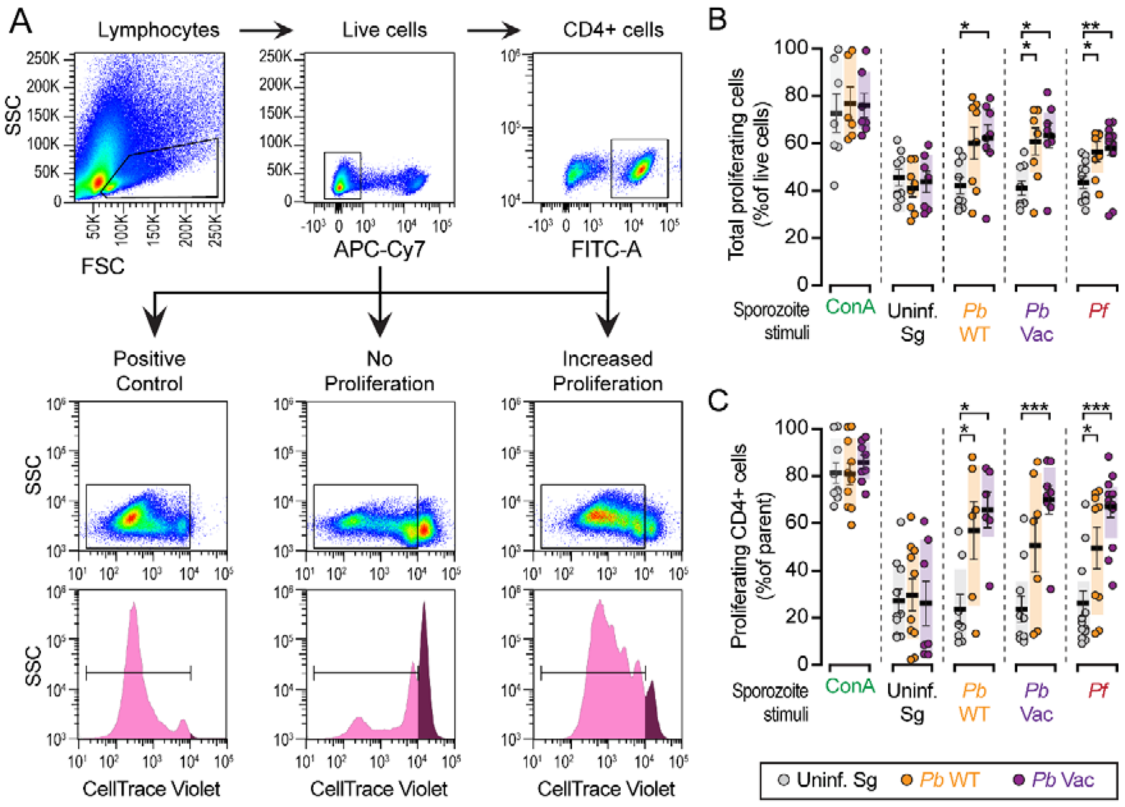

Fig. S9. Flow cytometry based assessment of cellular immune responses elicited upon immunization with *Pb*Vac.

(A) Schematic illustration of the flow cytometry-based strategy employed for assessment of cell proliferation. Incorporation of the dye cell-trace violet by nascent generation of proliferating cells was quantified relative to the overall number of cells present within the parent population; **(B)** Flow cytometry-based assessment of overall proliferation of lymphocytes isolated from the spleens of immunized rabbits upon stimulation with *Pb*WT, *Pb*Vac or *Pf* sporozoites. Stimulation with an extract of uninfected mosquito salivary gland material was used as negative control and sstimulation with Concanavalin A as a positive control. **(C)** Flow cytometry-based assessment of CD4^{+} T cells proliferation in the spleens of immunized rabbits following stimulation with different sporozoite stimuli; The boxes correspond to the 25th and 75th percentiles; the line and bars indicate mean of infection and standard error of the mean, respectively; *, p<0.05; **, p< 0.01; ***, p<0.001, as determined by by Kruskal-Wallis test, corrected with Dunn's multiple comparisons test.

Table S1. Number of predicted CD8^{+} T cell strong binding epitopes[a]

Molecules	*P. falciparum*		*P. berguei*		Shared by *Pf* and *Pb*
	Nuclear	Total[b]	Nuclear	Total	
Epitopes[c]	769076	771579	265459	771579	24171[e]
Proteins[d]	5515	5548	5027	5059	

[a] Epitopes with the top 0.5% of affinity binding prediction scores are considered strong binders. [b] Total numbers are based on the sum of epitopes for nuclear and organelle-encoded proteins. [c] Epitopes were predicted in the complete set of predicted proteins in *Pf* and *Pb*, consisting of 5548 and 5076 proteins, respectively; predictions were conducted with NetMHCpan, based on ten representative alleles of the HLA-A and -B supertypes (**Table S4**). [d] Total number of proteins containing predicted epitopes; virtually all proteins is each species contains one or more predicted strong binding epitopes. [e] The 24171 epitopes shared between species are encoded in 3371 *Pf* proteins and 3332 *Pb* proteins, of which 3223 are orthologous protein pairs (**Fig. SX**).

Table S2. HLA types and global frequencies used for CD8+ T Cell epitope predictions

HLA Type[a]	Supertype	Worldwide, Allele Count	Worldwide Frequency
A01:01	A01	358,665	11.07
A02:02	A02	632,158	19.52
A03:01	A03	305,012	9.45
A24:02	A24	308,925	9.54
B07:02	B07	269,570	8.32
B08:01	B08	214,823	6.63
B15:01	B62	125,296	3.87
B27:05	B27	72,771	2.25
B44:02	B44	171,229	5.28
B58:01	B58	71,025	2.19

[a] Frequency of A and B HLA types based on a worldwide survey of 3,239,247 and 3,240,802 individuals, respectively. Information compiled from The Allele Frequency Net Database (accessed on February 22, 2018).

Table S3. Primers for generation of DNA construct

DNA Construct	Primer	Primer sequences *	Restriction sites	fragment size (bp)	Description
**pL1988**	**7244**	ataagaat**gcggccgc**CAATTCATGATGAGAAAATTAGC	NotI	1,243	*Pf*CSP ORF R
	**7240**	gtgt**caccggcg**AGATGTGTTCTTTATCTAATTAAGG	SgrAI		*Pf*CSP ORF R
	**7169**	tat**cctgcagg**GTGATAGTGTAGATTTTTTTGTTTGAC	SbfI	1,519	*Pb*UIS4 5'-UTR pro., F
	**7170**	ataagaat**gcggccgc**AGACGTAATAATTATGTGCTGAAAGG	NotI		*Pb*UIS4 5'-UTR pro.R
	**7171**	cg**gatatc**TATAATTCATTATGAGTAGTGTAATTCAG	EcoRV	1,025	*Pb*UIS4 3'-UTR, F
	**7172**	ggcc**ggtacc**TTTCGCTTTAATGCTTGTCATC	KpnI		*Pb*UIS4 3'-UTR, R

* Red color: Restriction site sequence

Table S4. Primers for genotyping *Pb*Vac

Primer	Description	Primer sequences *
**1011**	***Pf*CSP F**	cccg**ctcgag**CGCCAATTCATGATGAGAAAATTAGC
**1012**	***Pf*CSP R**	ataagaat**gcggccgc**CTTTATCTAATTAAGGAACAAGAAGGATAATACC
**1080**	***Pb*5'UIS4 promoter integration F**	ACTGTTATATTTGGTGATGGAATGG
**1081**	***Pb*5' UIS4 promoter integration R**	TATACATCCACGGATGCATAGAAG
**1082**	***Pb*3' UIS4 UTR integration F**	AACAGTGAAATATAAATATGAATGGAAGCAG
**1083**	***Pb*3' UIS4 UTR integration R**	TTCAGTGAAATCGCAAACATAAGTATC
**1048**	**hDHFR-yFCU (+/-SM) F**	ATCATGCAAGACTTTGAAAGTGAC
**1049**	**hDHFR-yFCU (+/-SM) R**	CATCGATTCACCAGCTCTGAC

* Red color: Restriction site sequence
